# Prolonging genetic circuit stability through adaptive evolution of overlapping genes

**DOI:** 10.1093/nar/gkad484

**Published:** 2023-06-01

**Authors:** Jennifer L Chlebek, Sean P Leonard, Christina Kang-Yun, Mimi C Yung, Dante P Ricci, Yongqin Jiao, Dan M Park

**Affiliations:** Biosciences and Biotechnology Division, Lawrence Livermore National Laboratory, Livermore, CA 94550, USA; Biosciences and Biotechnology Division, Lawrence Livermore National Laboratory, Livermore, CA 94550, USA; Biosciences and Biotechnology Division, Lawrence Livermore National Laboratory, Livermore, CA 94550, USA; Biosciences and Biotechnology Division, Lawrence Livermore National Laboratory, Livermore, CA 94550, USA; Biosciences and Biotechnology Division, Lawrence Livermore National Laboratory, Livermore, CA 94550, USA; Biosciences and Biotechnology Division, Lawrence Livermore National Laboratory, Livermore, CA 94550, USA; Biosciences and Biotechnology Division, Lawrence Livermore National Laboratory, Livermore, CA 94550, USA

## Abstract

The development of synthetic biological circuits that maintain functionality over application-relevant time scales remains a significant challenge. Here, we employed synthetic overlapping sequences in which one gene is encoded or ‘entangled’ entirely within an alternative reading frame of another gene. In this design, the toxin-encoding *relE* was entangled within *ilvA*, which encodes threonine deaminase, an enzyme essential for isoleucine biosynthesis. A functional entanglement construct was obtained upon modification of the ribosome-binding site of the internal *relE* gene. Using this optimized design, we found that the selection pressure to maintain functional IlvA stabilized the production of burdensome RelE for >130 generations, which compares favorably with the most stable kill-switch circuits developed to date. This stabilizing effect was achieved through a complete alteration of the allowable landscape of mutations such that mutations inactivating the entangled genes were disfavored. Instead, the majority of lineages accumulated mutations within the regulatory region of *ilvA*. By reducing baseline *relE* expression, these more ‘benign’ mutations lowered circuit burden, which suppressed the accumulation of *relE-*inactivating mutations, thereby prolonging kill-switch function. Overall, this work demonstrates the utility of sequence entanglement paired with an adaptive laboratory evolution campaign to increase the evolutionary stability of burdensome synthetic circuits.

## Graphical Abstract

**Graphical Abstract:**
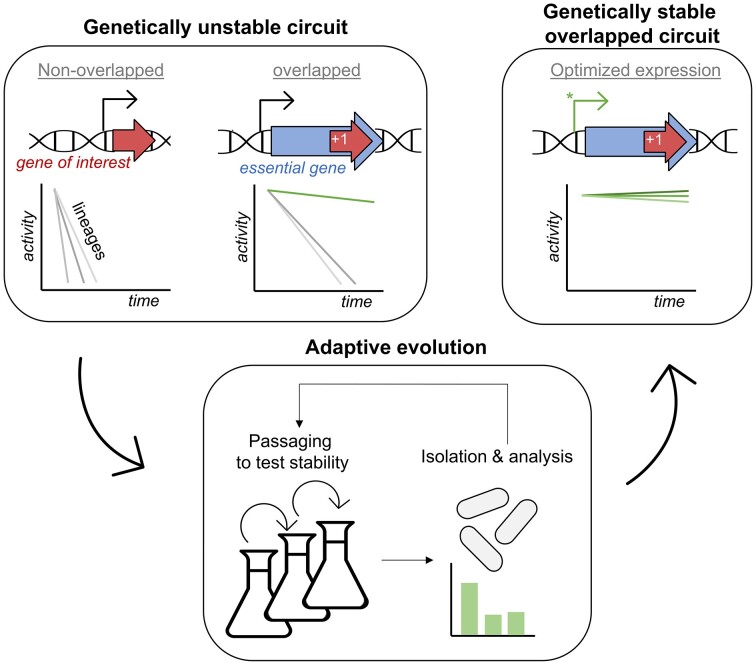

## INTRODUCTION

For the past several decades, synthetic biologists have sought to genetically engineer microorganisms for a wide range of applications including therapeutics discovery and delivery, drug manufacturing, agricultural yields, biofuel production, mineral extraction and waste degradation (1–8). For example, the microbial consortium that colonizes the rhizosphere of plant roots can be genetically engineered to enhance nutrient acquisition and drought resistance of agriculturally important crops (9,10). Such biotechnology applications require robust and stable expression of genetic circuits. Problematically, genetic circuit instability frequently originates from a fitness cost to the host due to leaky product toxicity (i.e. kill-switches) (11), host burden from adverse interactions with host components (12) or misallocation of resources (13–16). As a consequence, inactivating mutations accumulate and the resulting cells with disabled circuits rapidly outcompete the parent strain due to the relieved toxicity (17–20). For example, kill-switch circuits—which aim to control cell proliferation through regulated activity of a toxin—can fail when mutations arise that ablate the toxin's function, allowing for lineages harboring non-toxic circuits to quickly overtake the population (21). Therefore, the development of tools that mitigate genetic instability while maintaining circuit function is necessary and central to synthetic biology.

Prior efforts to improve DNA sequence fidelity have focused on reducing the background mutation rate (22,23), eliminating mutation-prone sequences (24), removing insertion sequence (IS) elements or avoiding them altogether through host selection (25,26), reducing burden of an engineered function (14) or increasing mutation surveillance and correction (27). However, the broad implementation of these genetic engineering efforts remains a challenge as it is difficult to predict which modifications are needed to achieve circuit stability in a given system *a priori*. Alternatively, laboratory evolution experiments have been used to optimize circuits by allowing for the unbiased selection of increased stability and performance (13,28)_._ Previous work has sought to engineer systems that allow adaptive evolution (29) to maintain and stabilize the function of burdensome circuits, such as linking the expression of a toxic gene to an essential function (25,30,31) or dividing maintenance and production of a toxic gene between different members in a consortium (32). However, many of these stabilizing systems are complex, rely on multiple levels of redundancy and have only been implemented in *Escherichia coli* (33,34). Thus, more generalizable and effective methods are needed for improving genetic circuit stability, especially for sequence regions that are prone to mutational inactivation.

Recently, approaches using gene overlaps have been developed to enhance sequence stability (35–37). Gene overlaps occur naturally in many biological systems, especially those with high mutation rates and compact genomes such as viruses (38–42). Gene overlaps impose constraints on sequences and their evolution given that mutations can impact the function of both genes involved (39,43–45). A pioneering method to accomplish synthetic gene overlap is gene entanglement in which two genes are synthetically encoded (‘entangled’) within the same DNA sequence but translated from different open reading frames (35). By entangling a gene-of-interest (GOI) with an essential gene, the evolution of the GOI can be constrained because mutations in the GOI may also be deleterious to the essential gene encoded in another frame. In one example in *E. coli*, entanglement of an amino acid biosynthetic gene *ilvA* with an essential gene *acpP* severely restricted the range of point mutations permissible within the overlap region of *ilvA* (35). While promising, the ability of synthetic gene overlaps to maintain genetic stability of engineered circuits remains untested.

Here, we demonstrate the use of sequence entanglement for improving genetic circuit stability, particularly for burdensome components that are prone to mutational inactivation. We assessed the feasibility of a proof-of-concept entanglement pair composed of a gene with high fitness cost (the toxin-encoding *relE*) and an essential gene (*ilvA*) to improve genetic stability of a toxin-based kill-switch circuit in the environmentally relevant microorganism *Pseudomonas protegens* Pf-5. Our findings provide insight into how gene entanglement alters the allowable landscape of mutations and evolutionary trajectory of synthetic circuits, and showcase the ability of sequence entanglement to enable the use of natural selection to isolate cells with reduced fitness burden and more stable kill-switch function.

## MATERIALS AND METHODS

### Strains and culture conditions

*Pseudomonas protegens* Pf-5 was routinely grown in Luria–Bertani (LB) medium supplemented with kanamycin (20 μg/ml), gentamicin (5 μg/ml) or tetracycline (25 μg/ml) when appropriate. Stellar Competent *E. coli* cells (Takara Bio) were routinely grown in LB medium supplemented with kanamycin (50 μg/ml), gentamicin (15 μg/ml) and carbenicillin (100 μg/ml) when appropriate. All strains were grown overnight either with shaking at ∼220 rpm or statically at either 30°C (for *P. protegens*) or 37°C (for *E. coli*), unless otherwise noted. M9 minimal medium (Sigma) was supplemented with 1 mM MgSO_4_, 100 mM CaCl_2_ and 20 mM glucose.

### Construction of mutant strains

**Figure 1.**
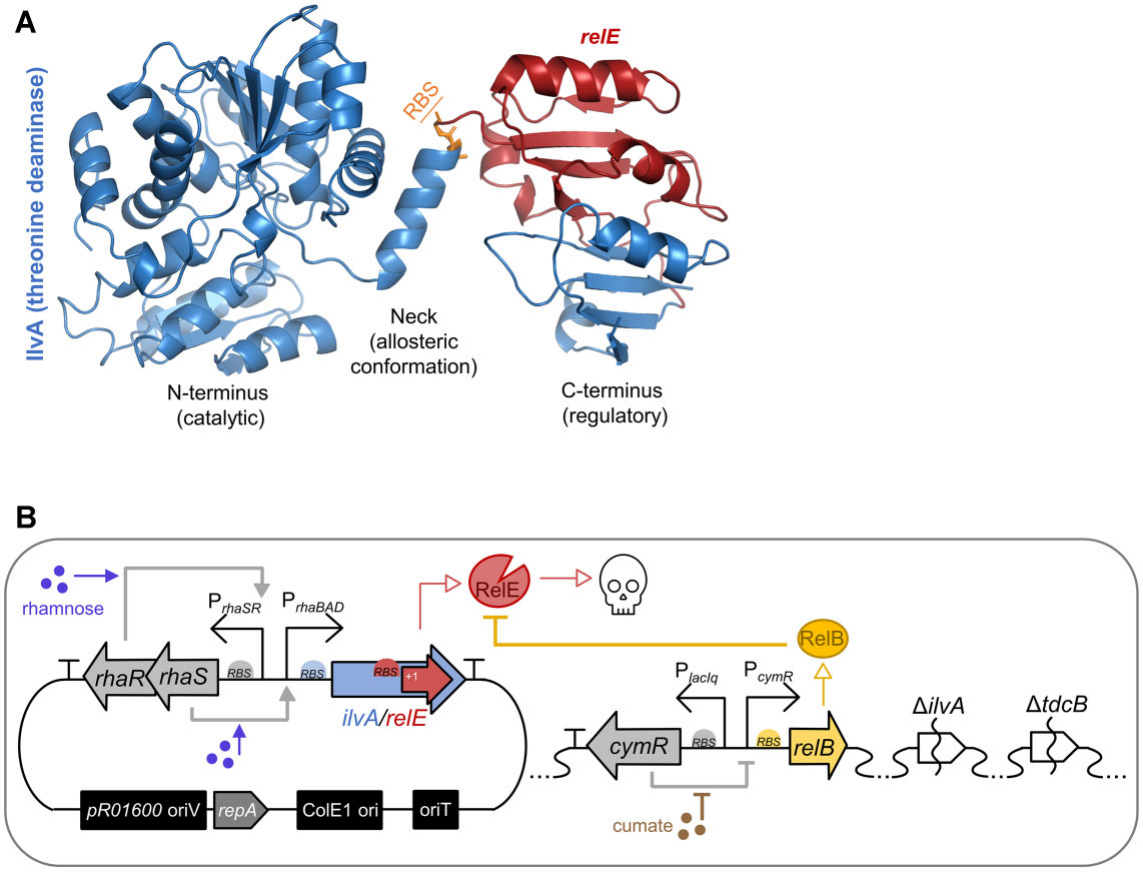
Structure and genetic sequence components of the *ilvA*/*relE* entanglement. (**A**) Structure of WT *E. coli* threonine deaminase (blue) with the location of the *relE* sequence within the +1 frame of the *ilvA*/*relE* entanglement highlighted in red. The protein domains of IlvA are labeled and the region of amino acid changes imparted by the RBS modifications in *ilvA*/*relE*^RBSX^ constructs are shown in orange. The Protein Data Bank (PDB) accession number is 1TDJ. Structural renderings were generated using PyMOL v. 2.3.4. (**B**) Diagram of genetic circuit components used in this study. *P. protegens* Pf-5 was made auxotrophic for isoleucine via Δ*ilvA* and Δ*tdcB* chromosomal deletions (right). Addition of cumate induces antitoxin expression by relieving CymR repression of chromosomal P*_cymR_*-*relB* (right), while addition of rhamnose increases expression of *ilvA*/*relE* (or related alleles) through activation of plasmid-borne RhaR and RhaS, which activate P*_rhaBAD_* (left).

Primers (Supplementary Table S1) were ordered from Integrated DNA Technologies (IDT), and constructs were amplified by polymerase chain reaction (PCR) with Q5 High Fidelity polymerase (NEB). For all vectors, cloning was completed using InFusion (Takara Bio), according to the manufacturer's instructions. RhaRS/P*_rhaBAD_* and CymR/P*_cymR_* (46) were added to the base vector [either pJUMP24-1A (47) modified with the T24 terminator downstream of the cloning site or the miniTn7PuC18 vector (48), respectively] prior to cloning. All plasmids were sequence verified by Elim Biopharm or SNPsaurus. pJUMP24-T24-P*_rhaBAD_* vectors were cloned into Stellar Competent *E. coli* using heat shock according to the manufacturer's instructions, and plasmids were maintained in LB medium with 50 μg/ml kanamycin. Vectors were transformed into *P. protegens* via electroporation as described previously (48) and maintained in LB medium with 20 μg/ml kanamycin unless otherwise noted. In the *P. protegens* parent strain used in this study, CymR/P*_cymR_*-*relB* (the antitoxin) was integrated within the chromosomal attTn7 site (48) such that *cymR* expression was driven by the *lacIq* promoter, resulting in constitutive expression of CymR (Figure 1). All overnight cultures were prepared from glycerol stocks and grown in the presence of 0.5 mM cumate to relieve CymR repression and induce the antitoxin *relB*, except where indicated. See Supplementary Table S2 for a detailed list of all mutant strains used throughout this study.

In-frame deletions of *ilvA* (PFL_5905) and *tdcB* (PFL_3098) in *P. protegens* were obtained by a two-step *sacB* counterselection procedure (49). Approximately 700 bp regions flanking the 5′ and 3′ regions of *ilvA* or *tdcB* were amplified using the primer sets described in Supplementary Table S1 and cloned into the HindIII- and BamHI-digested suicide plasmid pNPTS138 using InFusion cloning. The pNPTS138-based deletion plasmids were transformed into *E. coli* MFDpir and conjugated into *P. protegens* Pf-5. Primary integrants were selected on LB medium containing 25 μg/ml kanamycin. Counterselection for the second chromosomal cross-over event, resulting in gene deletion, was selected for by overnight growth in LB medium followed by plating on LB agar containing 3% sucrose. Deletions were confirmed by colony PCR and the Δ*ilvA* Δ*tdcB* strain was whole genome sequenced. The original *ilvA*/*relE* vector along with the vectors containing the RBS3 modification and vectors from CB2, CB3 and CB4 have been deposited in Addgene (plasmid number 201531–201535), where maps and sequences can be found.

### Assessment of growth in liquid culture

Cells were grown overnight in LB medium + kanamycin + 0.5 mM cumate and then washed twice in minimal medium. Cells were then diluted 1:25 in either LB or M9 minimal medium with kanamycin in Nunc™ Edge™ 96-Well flat bottom microplates (ThermoFisher). The following concentrations of inducers were added when indicated: 0.001% (w/v) rhamnose to induce the expression of P*_rhaBAD_* (50), 0.5 mM cumate to induce the expression of P*_cymR_*-*relB*, and 1 mM isoleucine. Assays were performed at 30°C with linear shaking, and growth was kinetically monitored by measuring OD_600_ on an Agilent HTX plate reader.

### Western blot analysis

To assess threonine deaminase (*3×FLAG-ilvA*) production, strains were grown overnight in LB containing kanamycin. Strains were subcultured the next day 1:100 in fresh LB medium supplemented with kanamycin and 0.001% (w/v) rhamnose, and grown with shaking for 6 h at 30°C. For each sample, cells were centrifuged and resuspended to an OD_600_ = 25.0 in B-PER™ Complete Bacterial Protein Extraction Reagent (Thermofisher) with 1 mM phenylmethylsulfonyl fluoride (PMSF) and 38 μg/ml lysozyme, and incubated at room temperature for 15 min. Samples were then mixed 1:1 with 2× sodium dodecylsulfate (SDS) sample buffer (220 mM Tris pH 6.8, 25% glycerol, 1.8% SDS, 0.02% Bromophenol Blue, 5% β-mercaptoethanol) and boiled for 10 min at 100°C. Then, 10 μl of each sample was separated on an Any kD™ Mini-PROTEAN® TGX™ Precast Protein Gel (BioRad). Proteins were electrophoretically transferred to a polyvinyldifluoridene (PVDF) membrane using a Trans-Blot® Turbo™ Transfer System (BioRad) and then incubated with the primary antibodies α-FLAG polyclonal mouse (1:12 000) and α-RpoA monoclonal mouse (1:1000) (Biolegend). The blots were washed and incubated with a α-rabbit and α-mouse secondary antibodies conjugated to horseradish peroxidase (HRP) and developed using Pierce ECL Western blotting substrate. Blots were imaged using a BioRad ChemiDoc instrument.

### Single time-point escape ratio

Single colonies of *P. protegens* Pf-5 were picked from LB agar supplemented with kanamycin and 0.5 mM cumate, and were inoculated in 3 ml of LB medium supplemented with kanamycin and 0.5 mM cumate, and grown with shaking at 30°C overnight. Cells were washed twice with minimal medium and serially diluted onto M9 minimal agar containing kanamycin, 0.001% rhamnose and 1 mM isoleucine with or without 0.5 mM cumate (i.e. permissive or non-permissive conditions, respectively). Plates were incubated statically at 30°C for ∼48 h and then single colonies were picked and patched onto minimal medium agar plates supplemented with kanamycin and 0.001% rhamnose either with or without 1 mM isoleucine. To calculate the escape ratio, the colony-forming units (CFU) per ml present on non-permissive medium was divided by the CFU/ml on permissive medium. For the condition without isoleucine, the ratio of colonies that survived on non-permissive plates without isoleucine was multiplied by the escape ratio from plates with isoleucine. To identify mutations causing escape, one colony per replicate was isolated from the patched plates for each growth phenotype; they could only grow either in the presence of isoleucine or under both conditions. The chromosomal P*_cymR_-relB* region of each colony was PCR amplified and the linear products were sequenced at Elim Biopharm or SNPsaurus. The plasmids of these colonies were purified via miniprep (Qiagen) and sequenced by SNPsaurus. A detailed list of mutations can be found in Supplementary Table S3.

### Long-term evolutionary stability experiments

Single colonies of *P. protegens* Pf-5 strains were picked from LB agar supplemented with kanamycin and 0.5 mM cumate, and were inoculated into 5 ml of the minimal medium supplemented with kanamycin, 0.5 mM cumate and 0.001% (w/v) rhamnose with or without 1 mM isoleucine, and grown with shaking at 30°C for ∼6.6 generations (∼24 h). Here, rhamnose was added to ensure sufficient *ilvA*/*relE*^RBS3^ expression for isoleucine production, and cumate was added to induce sufficient RelB expression to minimize the toxic effects of RelE expression. Cultures were passaged with a 1:100 dilution into fresh medium at 24 h intervals. To measure the escape ratio, cells were washed once with minimal medium and serially diluted onto M9 minimal agar containing the same inducers as when grown in liquid culture, except with or without 0.5 mM cumate (i.e. permissive or non-permissive conditions, respectively). Plates were incubated statically at 30°C for ∼48 h and then the CFU/ml was counted. The escape ratio was calculated as the ratio of colonies on plates without cumate relative to total viable colonies on plates with cumate. Escape ratio measurements were taken at each 24 h interval for 10 days (∼66 generations) or at a 5 day interval for 20 days (∼132 generations). The number of generations per day (*n*) was calculated as *n* = [log(*N*_t_) – log(*N*_o_)]/log(2), where *N*_t_ is the final OD_600_ and *N*_o_ is the initial OD_600_. To identify mutations causing escape, colonies were isolated from the final passage of each separate lineage selected on permissive plates with 0.5 mM cumate. The chromosomal P*_cymR_*-*relB* region of each colony was amplified and the linear products were Sanger sequenced at SNPsaurus. The plasmids of these colonies were purified via miniprep (Qiagen) and sequenced by SNPsaurus. A detailed list of mutations can be found in Supplementary Table S3.

### Whole-genome sequencing of evolved lineages

Whole-genome DNA library preparation and short read sequencing was performed by SeqCenter. Briefly, we grew clones from evolved lineages (Figure 5A, isolates 1–10 from both conditions) and an ancestor population (Supplementary Table S2; SJC072) to saturation overnight in LB medium and centrifuged 1 ml at 16 000 *g* for 1 min. These pellets were frozen and shipped overnight to SeqCenter, where whole-genome DNA extraction was performed (Zymo DNA Miniprep CAT# D3024), and sequencing libraries were created with an Illumina DNA Prep kit. Libraries were sequenced on an Illumina NovaSeq 6000 with 2 × 151 bp paired-end reads. Demultiplexed reads were provided by SeqCenter.

Libraries were quality and adapter trimmed using *trim-galore* (https://github.com/FelixKrueger/TrimGalore) with default parameters (51,52). To identify mutations in evolved lineages, we used *breseq* (53) run in default ‘consensus’ mode. To create a suitable reference genome that accounted for mutations present in all lineages at the beginning of the evolution experiment, we compared the *P. protegens* Pf-5 reference genome NC_004129 with our ancestor and verified the Tn*7* insertion sequence. Sequencing libraries from all evolved lineages were then compared with this updated reference using *breseq*. We manually verified the read evidence presented for each identified mutation and retained those with high-confidence predictions. All sequencing libraries are available at BioProject PRJNA970322. Whole-genome sequencing data for each isolate can be found in Supplementary Table S3.

### Green fluorescent protein (GFP) assay

Single colonies were grown in minimal medium containing kanamycin and 0.005% (w/v) rhamnose overnight at 30°C. The next day, cells were washed and resuspended to an OD_600_ = 1.0 in minimal medium. Fluorescence and OD_600_ was determined on an Agilent HTX plate reader with excitation at 500 nm and emission at 540 nm. Relative fluorescent units (RFU) are reported normalized to the OD_600_.

### Competition assay

Overnight cultures were grown in LB medium containing kanamycin and 0.5 mM cumate, were washed twice and were resuspended to a final OD_600_ of 1.0 in M9 minimal medium. Each culture was mixed in a 1:1 ratio with the parent strain. For each mixture, ∼10^5^ cells total were added to 5 ml of M9 minimal medium supplemented with 0.001% rhamnose and 0.5 mM cumate, and grown with shaking at 30°C for 48 h. CFU/ml was determined at initial inoculation and after 48 h by dilution plating on LB agar containing kanamycin supplemented with either gentamycin or tetracycline. Competing strains were discerned by growth on either gentamycin- or tetracycline-containing plates, as the parent strain was gentamycin resistant/tetracycline sensitive, and the other strains were gentamycin sensitive/tetracycline resistant. Competitive indices were calculated as the CFU ratio of the evolved strain (CB strains 2, 3 and 4) over the parent strain after growth for 48 h divided by the CFU ratio in the initial inoculum (54).

### Statistics

Statistical differences were assessed by Student's *t*-test or one-way analysis of variance (ANOVA) tests followed by either a Dunnett's or Tukey's multiple comparisons post-hoc test as indicated using GraphPad Prism software v. 9.5.0. For all escape ratio experiments, statistical analyses were performed on log-transformed data. All statistical comparisons can be found in Supplementary Table S4.

## RESULTS

### Post-entanglement modifications enhance functionality of the entanglement pair

In order to assess gene overlaps as a method for improving the sequence fidelity of synthetic gene circuits, we selected a previously developed but moderately functional entanglement pair in *E. coli* (35) and ported it into the soil microbe, *P. protegens* Pf-5. This organism was chosen because it is an attractive rhizophore-dwelling candidate for hosting bioengineered circuits due to its plant-promoting functions and lack of IS elements (55), which are known to contribute to circuit inactivation (13,18,56). The entanglement pair is comprised of a toxin (*relE*; 288 bp) embedded in the +1 frame of a conditionally essential gene (*ilvA*; 1542 bp). The gene *relE* encodes a mRNA-degrading endoribonuclease of the type II toxin–antitoxin family, and *ilvA* encodes threonine deaminase, which is required for isoleucine biosynthesis (57–59). Threonine deaminase is comprised of two primary domains—the N-terminal catalytic domain, which catalyzes the production of isoleucine, and the C-terminal regulatory domain, which provides positive and negative conformational feedback to the catalytic domain based on the availability of substrate (threonine and valine) and product (isoleucine) (60,61) (Figure 1A). The gene encoding the toxin *relE* was entangled into the C-terminal domain of *E. coli ilvA*. To accommodate a wild-type (WT) amino acid sequence for RelE, the *ilvA* sequence was significantly recoded with missense mutations (∼79% of entangled residues were altered) (Supplementary Figure S1). This pairing allows us to test whether an essential function (isoleucine biosynthesis) can improve the stability of a gene prone to mutational inactivation (*relE*).

**Figure 2.**
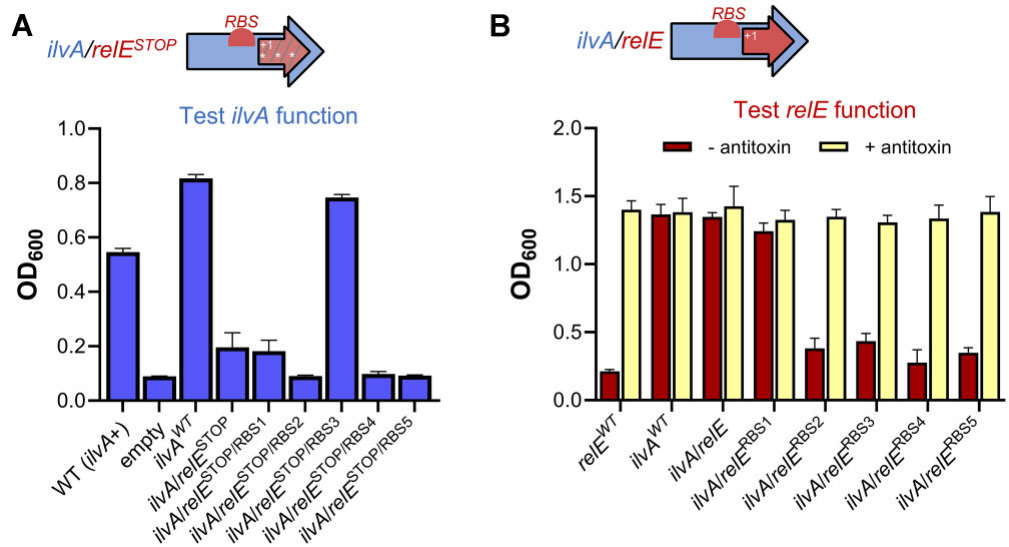
Internal RBS modifications improve functionality of *ilvA/relE*. (**A**) To probe *ilvA* function, strains harboring *ilvA*/*relE*^STOP^ alleles containing different strength RBSs were grown in minimal medium without isoleucine and without addition of rhamnose. In the diagram, stop codons in *relE* are represented by asterisks, and dashed lines indicate a non-functional *relE*. (**B**) Strains harboring *ilvA*/*relE* alleles with different strength internal RBSs were grown in rich medium to assess *relE* activity. To rescue growth, the antitoxin was induced by addition of cumate. For (A) and (B), strains harboring *ilvA*/*relE* vectors are listed in order of increasing internal RBS strength (see Supplementary Figure S5 for more details on RBS modifications). Growth is reported as OD_600_ after 15 h. Data are shown as the mean ± standard deviation (SD) of three independent replicates.

Previously, in *E. coli*, the *ilvA*/*relE* entangled pair was found to rescue the growth of an isoleucine auxotroph (Δ*ilvA*) in minimal medium, indicating that this entangled, recoded *ilvA* variant encodes an active enzyme (35). However, *ilvA*/*relE* minimally inhibited cell growth in rich medium, suggesting weak RelE activity (35). To test the functionality of the *ilvA/relE* entanglement construct in *P. protegens*, we first probed the ability of recoded *ilvA* to rescue growth of a strain made auxotrophic for isoleucine (Δ*ilvA*Δ*tdcB*; Supplementary Figure S2). To test recoded *ilvA* function separately from *relE* toxicity (35), we used an *ilvA*/*relE*^STOP^ allele that contains mutations that introduce multiple stop codons within the *relE* reading frame but are silent in the *ilvA* frame (35). A rhamnose-inducible promoter (P*_rhaBAD_*) was used to drive the expression of *ilvA/relE* (Figure 1B). Here, addition of rhamnose stimulates a regulatory cascade in which RhaS activates *rhaSR* expression and then RhaR activates the P*_rhaBAD_* promoter (62). Consistent with previously reported results in *E. coli*, *ilvA*/*relE*^STOP^ rescued the growth of a *P. protegens* Δ*ilvA*Δ*tdcB* strain but required a higher concentration of rhamnose compared with the non-entangled WT *ilvA* control (*ilvA*^WT^), suggesting that the product of entangled *ilvA* is partially functional (Figure 2A; Supplementary Figure S3). We next evaluated the activity of entangled *relE* by testing cell growth with chromosomally encoded *relB* antitoxin. A cumate-inducible promoter was used to regulate *relB* expression (P*_cymR_*-*relB*), wherein addition of cumate relieves CymR-mediated repression of the P*_cymR_* promoter (63–65) (Figure 1B). For the *ilvA*/*relE* strain, *relE* toxin induction failed to inhibit cell growth with or without *relB* antitoxin induction (Figure 2B; Supplementary Figure S4), whereas expression of non-entangled *relE*^WT^ severely inhibited cell growth in the absence of *relB* antitoxin (Figure 2B; Supplementary Figure S4). These results suggest that *relE* is not functional in the entanglement design.

We hypothesized that the lack of cellular toxicity by entangled *relE* is the result of poor translation, especially as no apparent ribosome-binding site (RBS) could be detected within *ilvA* upstream of the entangled *relE* reading frame (Supplementary Figure S5) (66). While the algorithm used to generate the entanglement designs (CAMEOS) takes into account protein fitness scoring while satisfying the protein co-encoding constraint, it does not automatically install an internal RBS to ensure translation of the internal gene embedded within the larger gene (Supplementary Figure S5) (35,67). We therefore sought to enhance RelE expression through post-hoc optimization of an internal RBS. Accordingly, we manually designed and engineered five *ilvA/relE*^RBS^ constructs with increasing predicted translation rates (67) upstream of the entangled *relE* start codon (Supplementary Figure S5). Growth assays revealed that four of the newly designed RBSs, namely *ilvA/relE*^RBS2^, *ilvA/relE*^RBS3^, *ilvA/relE*^RBS4^ and *ilvA/relE*^RBS5^, successfully recovered RelE activity and inhibited cell growth when the antitoxin was not induced (Figure 2B; Supplementary Figure S4). Only *ilvA/relE*^RBS1^ failed to improve toxicity, which was unsurprising as RBS1 had the lowest predicted translation rate (Supplementary Figure S5). Importantly, growth inhibition due to *relE* expression was rescued by induction of the *relB* antitoxin in each construct (Figure 2B; Supplementary Figure S4). This confirms that proper RelE activity was achieved through increased translation by the newly designed internal RBSs.

Given that optimizing the RBS strength of entangled *relE* also required non-synonymous point mutations within *ilvA*, we further tested whether the altered RBSs affect recoded *ilvA* function (Supplementary Figure S5). Induction of *ilvA/relE*^STOP/RBS1^, *ilvA/relE*^STOP/RBS2^, *ilvA/relE*^STOP/RBS4^ and *ilvA/relE*^STOP/RBS5^ rescued growth in minimal medium to a similar degree as the original *ilvA/relE*^STOP^ entanglement, which suggests that the amino acid changes imparted by the altered *relE* RBSs did not impair recoded IlvA function (Figure 2A; Supplementary Figure S3). Surprisingly, *ilvA/relE*^STOP^*^/^*^RBS3^ yielded a growth phenotype that closely mirrored the non-entangled *ilvA*^WT^; robust growth was observed in minimal medium without the addition of rhamnose. These results suggest that the RBS3 optimization increased either the activity or the abundance of entangled IlvA (Figure 2A, B). Western blots using functional 3×FLAG-tagged constructs showed that the RBS3 modification did not alter recoded IlvA protein abundance (Supplementary Figure S6A, B), implying an improvement in threonine deaminase enzymatic activity instead (see Supplementary Discussion). Overall, these data indicate that post-entanglement modification of the internal RBS improved the expression of the internally entangled gene (*relE*) with a positive impact on recoded IlvA functionality. Since RBS3 improved the toxicity of entangled *relE* while also yielding a growth phenotype nearly identical to that of *ilvA*^WT^, we focused our efforts on the *ilvA/relE*^RBS3^ design in follow-on experiments.

### Entanglement with *ilvA* enhances mutational robustness of a toxic genetic circuit

**Figure 3.**
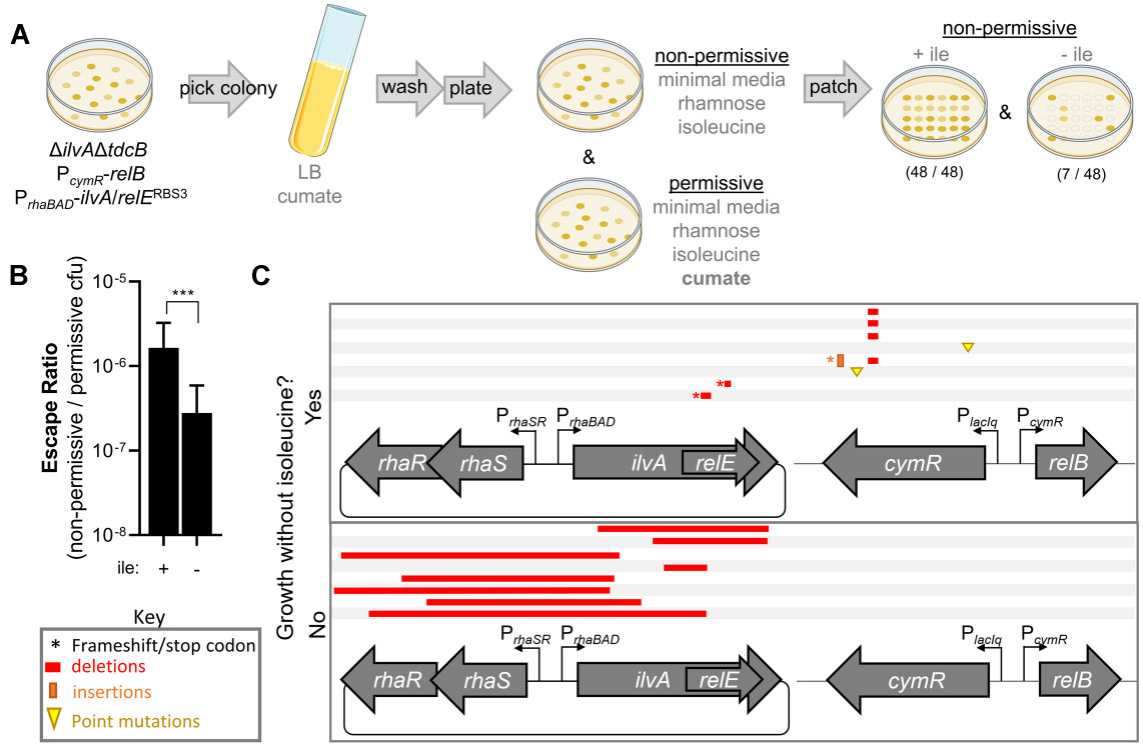
Escape ratio and the allowable landscape of mutations for *ilvA*/*relE*^RBS3^ following a shift from permissive to non-permissive conditions. (**A**) Diagram of the procedure used to determine the escape ratio. Single isolates of the strain Δ*ilvA*Δ*tdcB* P*_cymR_*-*relB* harboring a vector with *ilvA*/*relE*^RBS3^ were grown under permissive conditions (LB + cumate) and then plated for CFU on minimal medium under both non-permissive (toxin-induced) and permissive (anti-toxin-induced) conditions with isoleucine. For each replicate, 48 colonies were then patched from the non-permissive plate with isoleucine onto non-permissive plates either with or without isoleucine. (**B**) The escape ratio was used as a metric of *relE* function and was determined by dividing the CFU/ml on non-permissive medium by the CFU/ml on permissive medium with isoleucine before patching. This was then multiplied by the proportion of colonies that survived on the patched plates either with isoleucine (+ile) or without isoleucine (–ile), respectively. An example of this proportion is seen in (A). Data are shown as the mean ± SD of eight independent replicates. Comparisons were made by Student's *t*-test. ***P < 0.001. (**C**) Schematic showing the types of mutations present in toxin escape colonies isolated from non-permissive patch plates without (top) or with isoleucine supplementation (bottom).

Next, we sought to determine whether *ilvA/relE*^RBS3^ can increase the robustness of the embedded toxin to inactivating mutations. Because sequence entanglement imposes constraints on the evolution of both genes in the entangled pair, we expect that most mutations that arise in *relE* will also be deleterious to the function of recoded *ilvA*. Thus, we expect that growing the *ilvA*/*relE*^RBS3^ strain under conditions that require *ilvA* function would effectively protect *relE* from accruing inactivating mutations. To test this, we employed a toxin escape assay by growing the *ilvA*/*relE*^RBS3^ strain without selective pressure (rich medium, + antitoxin) and then plating on non-permissive (+ toxin, – antitoxin) and permissive (+ toxin, + antitoxin) minimal medium (Figure 3A). In the presence of isoleucine, we found that a strain harboring *ilvA*/*relE*^RBS3^ experienced an escape ratio (CFU under non-permissive conditions/CFU under permissive conditions) of ∼10^−6^, which is comparable with previous observations when *relE* is expressed in *Pseudomonas* (21) (Figure 3B). However, in the absence of isoleucine, the escape ratio is reduced by ∼7-fold, indicating that entanglement can lower the frequency of the population which accumulates RelE-inactivating mutations (Figure 3B). Sequencing the surviving colonies from non-permissive plates revealed that the majority of colonies on plates with isoleucine incurred mutations within the P*_rhaBAD_* promoter and RhaR/RhaS regulators of *ilvA*/*relE*^RBS3^, or large internal truncations that span across the entanglement region (Figure 3C). However, colonies from plates without isoleucine acquired no mutations in the promoter or regulators of *ilvA*/*relE*^RBS3^ (Figure 3C). Instead, these constructs harbored mutations in either the antitoxin regulator CymR or within the *relE* gene (small deletions) (Figure 3C). These more ‘benign’ mutations probably relieve RelE toxicity by increasing RelB antitoxin expression through reduced CymR activity or by directly ablating the catalytic activity of RelE. No mutations solely affecting IlvA activity were observed from plates without isoleucine, supporting the idea that there was selective pressure to maintain functional IlvA under this condition.

The finding that small deletions causing frameshifts within the *relE* entangled region failed to ablate threonine deaminase function raised the question of whether the C-terminal domain of IlvA is dispensable for isoleucine biosynthesis in *P. protegens*. To test this, we generated a truncation of *ilvA* just upstream of the entanglement position and the modified internal RBS that removes the entire C-terminus of IlvA (*ilvA*^ΔH322-G514^) (Supplementary Figure S1), and assessed whether this construct could support growth in minimal medium. We found that *ilvA*^ΔH322-G514^ complemented the isoleucine auxotrophy but exhibited a slower growth rate relative to *ilvA*^WT^ (Supplementary Figure S7A), suggesting that the C-terminal regulatory domain of IlvA may be important for optimal fitness even though it is not essential for isoleucine production in *P. protegens*. To test this further, we conducted a competitive growth assay (68) between *ilvA*^ΔH322-G514^ and either *ilvA*^WT^ or *ilvA*/*relE*^STOP/RBS3^. When grown in co-culture in the presence of rhamnose, we found that *ilvA*^WT^ and *ilvA*/*relE*^STOP/RBS3^ displayed an ∼17-fold and ∼5-fold competitive advantage, respectively, over *ilvA*^ΔH322-G514^, although the *ilvA*/*relE*^STOP/RBS3^ difference was not statistically significant (Supplementary Figure S7B). When grown in the absence of rhamnose, this effect was even more pronounced, where *ilvA*^WT^ and *ilvA*/*relE*^STOP/RBS3^ displayed a statistically significant ∼117-fold and ∼20-fold competitive advantage over *ilvA*^ΔH322-G514^, respectively (Supplementary Figure S7B). This result suggests that there is selective pressure to maintain the C-terminal regulatory domain of IlvA, even when redesigned to accommodate *relE* entanglement. However, the accumulation of polar mutations in this region during non-permissive conditions suggests that *relE* expression in the absence of the antitoxin is more deleterious to cell fitness than disruption of the C-terminal IlvA domain. Nevertheless, since mutations within *relE* were observed less frequently than those occurring within the *ilvA* regulatory elements, the data suggest that this entanglement design increases the mutational robustness of *relE* by protecting it from the most common inactivating mutations.

### Entanglement enhances long-term evolutionary stability of a toxic circuit

**Figure 4.**
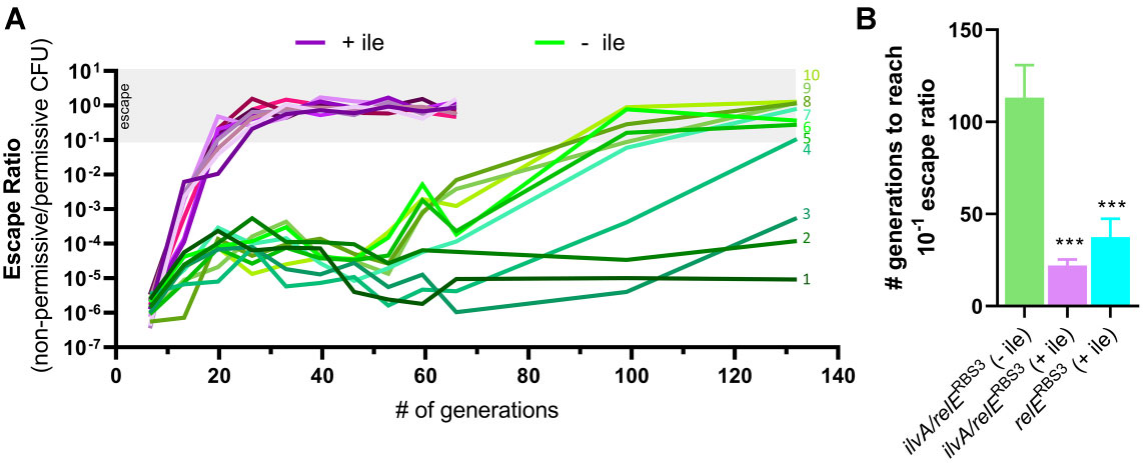
The *ilvA*/*relE*^RBS3^ entanglement increases the evolutionary stability of *relE*. (**A**) Independent lineages of Δ*ilvA*Δ*tdcB* P*_cymR_*-*relB* harboring *ilvA*/*relE*^RBS3^ on a vector were grown in minimal medium with rhamnose (to induce *ilvA*/*relE*^RBS3^) and cumate (to induce *relB* antitoxin) in the presence (purple) or absence (green) of isoleucine. Each day (∼6.6 generations) the cultures were diluted 1:1000 in fresh medium and plated for CFU in toxin-permissive and non-permissive conditions, and an escape ratio was calculated. After 10 days, measurements were taken less frequently (every 5 days). Passaging in medium with isoleucine was discontinued at ∼66 generations when the population was stabilized with a near 100% escape ratio. Each condition is represented by 10 independent lineages which started from single colonies and are numbered in order of increasing final escape ratio. The gray bar indicates a toxin escape ratio ≥ 10^−1^ (10%). (**B**) Average number of generations elapsed when the escape ratio exceeded 10^−1^ (i.e. 10%, gray bar in A). Raw data for *relE*^RBS3^ can be found in Supplementary Figure S9. The result for *ilvA*/*relE*^RBS3^ grown without isoleucine is an underestimate as three lineages from this condition never reached 10% escape and were excluded from the calculation. Bar graph data are shown as the mean ± SD. Asterisks directly above bars denote comparisons with the *ilvA*/*relE*^RBS3^ without isoleucine condition (– ile). Comparisons were made by one-way ANOVA with Tukey’s post-hoc test. ***P < 0.001.

The finding that entanglement with *ilvA* can protect *relE* from certain inactivating mutations suggested that *ilvA/relE*^RBS3^ may preserve a low toxin escape ratio over time and improve kill-switch stability. Previous deployments of *relE* in a kill-switch circuit showed that *relE* is inherently unstable and is rapidly inactivated by mutations during serial passaging even under permissive conditions (i.e. with antitoxin expression) (21). This may be due to stochastic fluctuation in the toxin/antitoxin levels such that toxin levels exceed antitoxin levels at sufficient frequency to create selective pressure against *relE* toxin expression (69,70). We hypothesized that (i) mutations that inactivate entangled *relE* function will rapidly outcompete the original design due to fitness burden imposed by RelE toxicity, and (ii) growing these lineages in the absence of isoleucine may effectively slow down the accumulation of *relE*-inactivating mutations within *ilvA*/*relE*^RBS3^. Accordingly, we assessed the stability of the P*_rhaBAD_*–*ilvA*/*relE*^RBS3^ circuit over 100+ generations in medium with or without isoleucine. We conducted serial passaging of 20 independent lineages of Δ*ilvA*Δ*tdcB* P*_cymR_*-*relB* carrying the P*_rhaBAD_*-*ilvA*/*relE*^RBS3^ vector under permissive conditions (+ toxin, + antitoxin). To determine the stability of the construct, each lineage was plated for CFU after each passage on both permissive (+ toxin, + antitoxin) and non-permissive (+ toxin, – antitoxin) medium supplemented with or without isoleucine in accordance with the condition in which they were passaged. This escape ratio is used as a proxy for *relE* activity. All *ilvA*/*relE*^RBS3^ lineages grown with isoleucine saw a dramatic increase in the escape ratio during the first ∼30 generations (from 10^−6^ to 10^−1^) (Figure 4A). In the following ∼40 generations, the escape ratio remained between 10^−1^ and 10^0^, indicating that *relE* was rendered non-functional in nearly the entire population (Figure 4A). We also probed the stability of non-entangled *relE*^RBS3^, which is driven by the same RBS3 modification and displayed comparable toxicity to *ilvA*/*relE*^RBS3^ (Supplementary Figure S8). All lineages of the non-entangled *relE*^RBS3^ reached an escape ratio of ≥ 10^−1^ at ∼40 generations (Supplementary Figure S9). This result confirms that when passaged with isoleucine, kill-switch circuits using *relE*^RBS3^ and *ilvA*/*relE*^RBS3^ are highly unstable.

In contrast to medium with isoleucine, all *ilvA*/*relE*^RBS3^ lineages passaged without isoleucine (a condition that selects for IlvA function) exhibited a more gradual increase in their escape ratio. A slight increase in escape ratio from 10^−6^ to 10^−4^ was observed for all lineages within the first ∼20 generations, before leveling off for the next ∼30 generations (Figure 4A). At ∼50 generations, a divergence in evolutionary trajectory took place. Seven of the lineages experienced a clear upward trend, reaching an escape ratio ≥ 10^−1^ by ∼113 generations (Figure 4A lineages 4–10; 4B green bar). However, the escape ratio for three of the lineages passaged without isoleucine remained low (∼10^−5^) even after 132 generations (Figure 4A, lineages 1–3). This marks a significant improvement over the *ilvA*/*relE*^RBS3^ and *relE*^RBS3^ lineages grown with isoleucine, which reached an average escape ratio of 10^−1^ by ∼22 generations and ∼37 generations, respectively (Figure 4B). Together, these data show that *relE* is more genetically stable and less susceptible to inactivating mutations when entangled with *ilvA* and passaged under conditions in which *ilvA* is required for growth.

### Entanglement alters the landscape of allowable mutations

**Figure 5.**
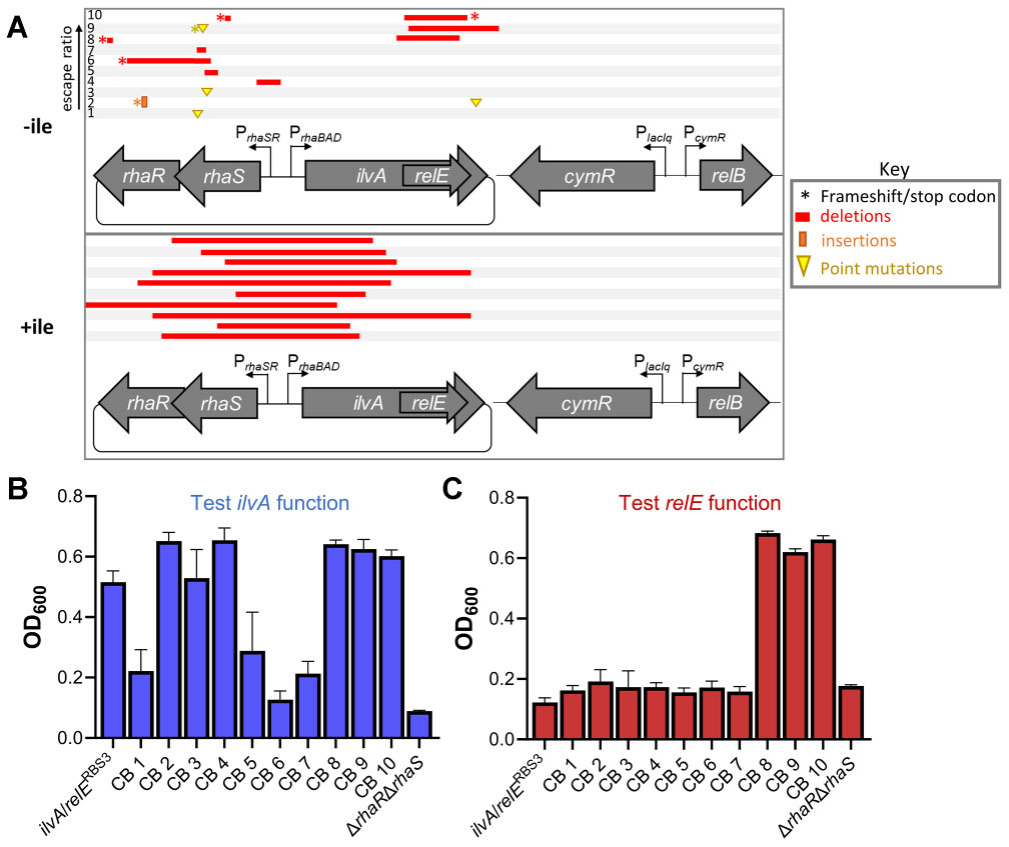
The *ilvA*/*relE*^RBS3^ entanglement alters the allowable landscape of mutations and maintains the function of both genes. (**A**) Schematic of the types of mutations present in colonies isolated from each lineage of the long-term evolutionary stability assay grown either with or without isoleucine. A single colony was isolated from each lineage under permissive conditions after the final passage. Mutations were identified by sequencing the entire vector and the *P_cymR_-relB* chromosomal region. Sequencing results from each isolate are listed in order of increasing final escape ratio seen in Figure 4A (isolates 1–10). The isolated vectors were transformed into a clean genetic background (Δ*ilvAΔtdcB P_cymR_-relB*) and were grown in (**B**) minimal medium without isoleucine and with addition of rhamnose and cumate to probe *ilvA* function or in (**C**) minimal medium with isoleucine and rhamnose and without cumate to test *relE* toxicity. For comparison with these clean background (CB) strains, the original parent strain (*ilvA*/*relE*^RBS3^) and a strain in which the regulators *rhaR* and *rhaS* are deleted from the vector (Δ*rhaR*/*rhaS*) are included. Growth is reported as OD_600_ after 15 h. Data are shown as the mean ± SD of three independent replicates.

To decipher the genetic basis for circuit stabilization by sequence entanglement, we sequenced the entire P*_rhaBAD_*-*ilvA*/*relE*^RBS3^ plasmid and P_*cymR*_-*relB* region from one colony randomly selected from the final passage of each lineage grown on a permissive plate (named isolates 1–10, Figure 4A). While we recognize that a single colony does not represent the entire population, it provides an indication of the types of mutations selected for under these growth conditions. For all lineages grown with isoleucine, large deletions were observed in the plasmid spanning the *rhaS* and/or *rhaR* regulatory genes and the *ilvA*/*relE*^RBS3^ entanglement region which undoubtedly disrupted the function of both the toxin and recoded IlvA (Figure 5A). This mutation pattern is similar to our findings from the single time point escape assay in which escape mutants were sequenced from medium containing isoleucine (Figure 3C). In contrast, such large and disruptive deletions were not observed in any of the lineages grown without isoleucine, indicating that the selective pressure to maintain IlvA function alters the landscape of allowable mutations in the circuit (Figure 5A). The colonies from the three lineages with the highest final escape ratio in the absence of isoleucine (Figure 4A; isolates 8, 9 and 10) contained a deletion of the entire *relE* gene (Figure 5A), which is an expected failure mode for this entanglement given the non-essentiality of the C-terminal portion of IlvA (Supplementary Figure S7). The other isolates from lineages grown without isoleucine contained point mutations or smaller insertions and deletions in the genes encoding the regulators *rhaR* and *rhaS* (Figure 5A). None of the isolates contained a mutation in the chromosomal P*_cymR_*-*relB* region (Figure 5A; Supplementary Table S3). We posit that these lineages, derived through adaptive evolution, led to improved circuit expression levels by reducing the burden of RelE while maintaining sufficient IlvA production. Therefore, we examined the vectors from these isolates to determine how the mutations may impact *ilvA*/*relE* activity.

We first assessed the toxicity of entangled *relE* and the function of recoded *ilvA* for the colonies isolated from the final passage of each lineage grown without isoleucine (isolates 1–10). When grown in minimal medium in permissive conditions (+ toxin, + antitoxin) without isoleucine, each isolate exhibited a shorter lag phase and a similar density after 15 h of growth compared with the parent strain (Supplementary Figure S10A, B), indicative of a functional IlvA. Next, we assessed entangled *relE* function by growing the strains in minimal medium under non-permissive conditions (+ toxin, – antitoxin) supplemented with isoleucine. We found that colonies isolated from lineages 8, 9 and 10 grew unimpeded, indicating a loss of *relE* activity (Supplementary Figure S10C, D). In contrast, isolates 1–7 exhibited a significant growth defect, indicative of functional *relE* (Supplementary Figure S10C, D). This result demonstrates that *relE* toxicity was preserved in at least a subset of the population for lineages 1–7 (Figure 4A).

To ascertain the role of the mutations in the evolved *ilvA*/*relE*^RBS3^ vectors in preserving fitness and maintaining or diminishing toxicity, we purified plasmids from the isolated colony of each lineage grown without isoleucine (i.e. from isolates 1–10) and transformed them into a clean genetic background (*P. protegens* Δ*ilvA*Δ*tdcB* P*_cymR_*-*relB*) (named CB strains 1–10). We then independently assessed the isoleucine auxotrophy and *relE* toxicity of these new CB strains, as described above. These data showed that CB 8, 9 and 10 supported growth in both permissive and non-permissive conditions, which confirmed that the deletion mutations in these vectors inactivated RelE while maintaining a functional IlvA (Figure 5B, C). Similar to the original isolates, CB 1–7 also displayed diminished growth under non-permissive conditions, which suggests that these vectors all contained functional *relE* (Figure 5C). While there was a delay in the growth-inhibitory effect of *relE* compared with the parent strain (Supplementary Figure S11A), the final OD_600_ for all CB strains with vectors from isolates 1–7 was similar to that of the parent *ilvA*/*relE*^RBS3^ strain (Figure 5C; Supplementary Figure S11A). This result demonstrates that the mutations in the *rhaR*/*rhaS* regulator region of CB 1–7 still permitted sufficient entangled RelE expression.

To probe the function of recoded IlvA in these vectors, we grew CB strains 1–7 in permissive conditions in the absence of isoleucine, which revealed that all seven vectors with functional *relE* supported some growth in the clean background, although there were notable differences in the lag phase of growth (Supplementary Figure S11B). CB strains 1, 5, 6 and 7 appeared more compromised in their ability to make isoleucine, as evidenced by an extended lag phase and lower OD_600_ after 15 h of growth compared with the parent strain (Figure 5B; Supplementary Figure S11B). This is in contrast to the results from the original colony isolates which showed that all strains can grow in minimal medium under permissive conditions (Supplementary Figure S10A, B). Thus, we suspected that there may be compensatory mutations elsewhere in the chromosome that contribute to isoleucine biogenesis in the original colonies isolated from lineages 1, 5, 6 and 7. Whole-genome sequencing analysis was completed on all isolates and revealed that colonies from lineages 1, 5 and 6 each contained mutations in global regulators, which we suspect may contribute indirectly to overcoming isoleucine auxotrophy, perhaps through pleiotropic effects that alter *ilvA*/*relE* expression or activity (Supplementary Table S3) (71,72). No chromosomal mutations were observed in isolate 7 and the reason for the growth difference in minimal medium between isolate 7 and the CB 7 strain remains unclear. In contrast, CB 2, 3 and 4 yielded robust growth compared with the parent construct, and CB strains 2 and 4 supported a shorter lag phase (Figure 5B; Supplementary Figure S11B). The mutations in CB 2, 3 and 4 include a 1 bp insertion in *rhaR* (*rhaR*^L128+1bp^), a missense point mutation in *rhaS* (*rhaS*^W190G^) and a deletion of the entire P*_rhaSR_* promoter including the first 15 bp of *rhaS* (*rhaS*^ΔM1-H5^/P*_rhaSR_*^Δ179bp^), respectively. With these mutations, it is possible that RhaS is still partially functional and can activate P*_rhaBAD_*. In contrast, CB strains 1, 5, 6 and 7 contain vectors with mutations which probably impact RhaS activity and more severely affect the expression of *ilvA*/*relE*^RBS3^: CB 1 has a substitution in the *rhaS* DNA-binding domain (*rhaS*^F254L^) that has been shown to be critical for RhaS activity (73), and CB 5, 6 and 7 harbor deletions within the DNA-binding domain of RhaS (Figure 5A). CB 2 also accumulated a point mutation in the IlvA C-terminus (*ilvA*^G455C^) downstream of entangled *relE*, which is not expected to impact RelE function. Due to the growth phenotypes observed among these CB strains, we hypothesize that the mutations in CB 2, 3 and 4 reduce the strength of the P*_rhaBAD_*-*ilvA*/*relE*^RBS3^ expression to a level compatible with functional IlvA production and reduced RelE burden under permissive conditions, while still preserving RelE toxicity under non-permissive conditions.

### Sequence entanglement allows for evolution to optimize circuit expression, which improves fitness and genetic stability

**Figure 6.**
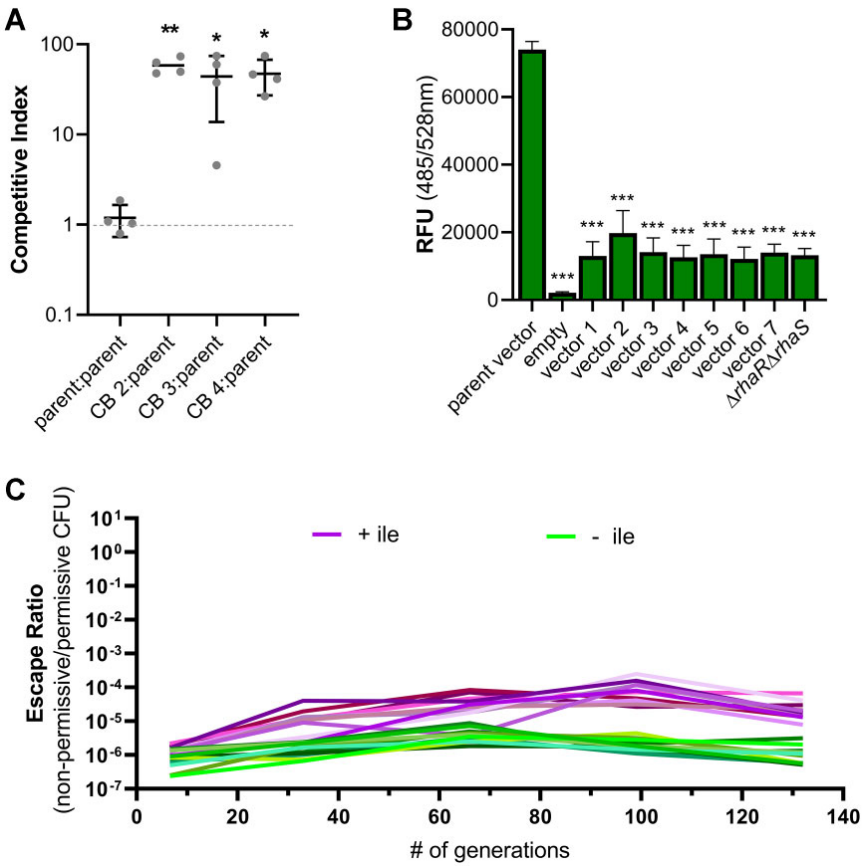
Sequence entanglement allows for circuit optimization through adaptive evolution. (**A**) Competition assay in which the parent strain *ilvA*/*relE*^RBS3^ was grown in a 1:1 co-culture with clean background strains harboring vectors isolated from the listed lineages (CB 2–4). Strains were differentially marked with chromosomally integrated antibiotic cassettes, tetracycline and gentamicin. The competitive index was calculated as the CFU ratio of mutant/parent after growth for 48 h divided by the CFU ratio of the mutant/parent in the initial inoculum. (**B**) Assay of promoter activity for vectors isolated from lineages 1–7 of the long-term evolutionary stability assay. The *gfp* gene was cloned downstream of the promoter of each vector, replacing *ilvA*/*relE*, and strains were grown in minimal medium overnight with rhamnose. RFU is reported relative to OD_600_. (**C**) Independent lineages of CB 2—a clean genetic background (Δ*ilvA*Δ*tdcB* P*_cymR_*-*relB*) strain harboring the *ilvA*/*relE*^RBS3^ vector isolated from the original lineage 2 in Figure 4B—were grown in minimal medium with rhamnose (to induce *ilvA*/*relE*^RBS3^) and cumate (to induce antitoxin) in the presence (purple) or absence (green) of isoleucine. Every 5 days (∼33 generations) the cultures were diluted 1:1000 in fresh medium and plated for CFU under toxin-permissive and non-permissive conditions, and an escape ratio was calculated. Each condition is represented by 10 independent lineages which started from single colonies grown on permissive medium. Data for (A) and (B) are shown as the mean ± SD of 3–4 independent replicates. Asterisk(s) directly above data denote comparisons with the parent condition. Comparisons were made by one-way ANOVA with Dunnet's (A) or Tukey's (B) post-hoc test. ***P < 0.001, **P < 0.01, *P < 0.05.

To determine if the mutations in CB 2, 3 and 4 improve cellular fitness compared with the original parent strain, we employed another competitive growth assay (68). We hypothesized that if the vectors from isolates 2, 3 and 4 imparted a competitive growth advantage to their respective host strains, then they should outcompete the parent strain if grown together. Indeed, a competitive growth assay, in which the original P*_rhaBAD_*-*ilvA*/*relE*^RBS3^ strain was grown in co-culture with CB 2, 3 or 4, revealed a competitive index between ∼37- and ∼51-fold greater than that of the parent strain after 13 generations of growth in permissive conditions (Figure 6A). These data reveal that the mutations in the P*_rhaBAD_* promoter of CB 2, 3 and 4 probably optimize the expression of their respective *ilvA*/*relE*^RBS3^ circuit and impart a significant growth advantage compared with the parent strain.

We next sought to understand how the accumulated mutations affect circuit expression and by what mechanism these mutations are improving fitness. Given the delay in the onset of *relE* toxicity with the vectors from isolates 1–7 (Supplementary Figure S11A) and the fact that each vector had mutations in *rhaR*/*rhaS* (Figure 5A), it seemed likely that these mutations reduced P*_rhaBAD_*-*ilvA*/*relE*^RBS3^ expression. To test this, we replaced *ilvA*/*relE*^RBS3^ with *gfp* in the plasmids from isolates 1–7 (named vectors 1–7) and evaluated GFP expression as a readout for promoter strength. Since many of these vectors contained mutations in *rhaR* and *rhaS* that we might expect to ablate activity of these regulators (i.e. deletions, frameshifts and point mutations), we also assessed the activity of a vector in which *rhaR* and *rhaS* were deleted (Δ*rhaR*/*rhaS*) for comparison. Interestingly, this analysis demonstrated that all seven vectors exhibited lower fluorescence compared with the original parent vector but were indistinguishable from the Δ*rhaR*Δ*rhaS* vector control (Figure 6B). We expected that GFP induction might be slightly higher in vectors 2, 3 and 4 because the mutations in these constructs still allowed for robust growth in minimal medium in a clean genetic background (Figure 5B), in contrast to a strain harboring a Δ*rhaR*Δ*rhaS* vector which did not support growth; however, no statistical difference in fluorescence was observed between vectors 1–7 (Figures 5B and 6B). It is possible that this *gfp* assay was not as sensitive as the growth-based assays in distinguishing between the low levels of induction observed in these vectors. Nevertheless, these data suggest that the mutations within the RhaR and RhaS regulators of each evolved lineage reduce P*_rhaBAD_*-*ilvA*/*relE*^RBS3^ expression, and this reduced expression is likely to be responsible for the delayed onset of *relE*-mediated growth inhibition in CB 1–7 strains (Supplementary Figure S11A) and the improved fitness observed in CB strains 2, 3 and 4 (Figure 6A). This result is consistent with previous findings that recommend lowering the expression of a toxic circuit for maintaining cellular fitness and circuit function (14,20,31,74,75).

We thus considered the intriguing possibility that mutations found in CB 2, 3 and 4 have decreased the pressure to select for mutations that abolish entangled *relE* toxicity, thereby enhancing the mutational stability of the toxin *relE* (76). To test this, CB 2 was chosen for passage over the course of 100+ generations under the same conditions as the initial long-term stability experiment in Figure 4A. Remarkably, all the CB 2 lineages grown under conditions with or without isoleucine exhibited a low and stable escape ratio, never reaching higher than 10^−4^ (Figure 6C). This is in sharp contrast to the rapid escape observed with the original parent strain during growth with isoleucine (Figure 4A). The CB 2 lineages passaged without isoleucine maintained an escape ratio between ∼10^−7^ and 10^−5^ over ∼132 generations. When passaged with isoleucine, CB 2 lineages are also quite stable and displayed only an ∼50-fold increase in escape ratio over the same time frame (Figure 6C). Together, these results suggest that the vector from the original isolate 2 (with the *rhaR*^L128+1bp^ insertion) imparts greatly enhanced *relE* long-term stability compared with the original parent vector (Figures 4A and 6C). This implies that even under conditions in which *ilvA* is not required for growth, the basal toxicity of *ilvA*/*relE*^RBS3^ from CB 2 does not impart a meaningful fitness defect under permissive conditions. Therefore, mutations that lowered the expression of *ilvA*/*relE*^RBS3^ appear to strike a balance between lessening *relE* toxicity while still maintaining *ilvA* function. In conclusion, these data demonstrate that sequence entanglement paired with adaptive evolution can optimize circuit function for prolonged evolutionary stability.

## DISCUSSION

When there is no selective pressure to maintain the function of a burdensome genetic circuit, loss-of-function mutations tend to accumulate in the circuit and cells with broken circuits overtake the population (77,78). The development of mechanisms to stabilize genetic circuits is an important step in deploying engineered biological systems for human benefit. The data presented here shed light on the utility of sequence entanglement to increase the long-term evolutionary stability of costly gene circuits in an environmentally relevant microbe. Below, we discuss key findings from the model *ilvA*/*relE* entanglement pair examined in this study.

Despite having *relE* entangled within a non-essential region of *ilvA* (the C-terminal regulatory domain), the *ilvA/relE*^RBS3^ entanglement was capable of stabilizing the toxin RelE by protecting against the most common circuit-inactivating mutations (i.e. large deletions across the P*_rhaBAD_* promoter and *ilvA/relE*). As such, this result is conceptually similar to that achieved with the Riboverlap method (37), where a partial gene overlap is created by inserting a translation initiation site for an essential gene within an upstream GOI, which achieves GOI protection against a subset of the mutational spectrum (e.g. frameshifts within the overlap region and promoter-inactivating mutations). An *in silico* analysis predicted that this Riboverlap approach would increase the temporal stability of the GOI under conditions in which the protected class of mutations are the most frequent inactivating mutations, but this result was not tested experimentally (37). In this study, we empirically demonstrate that entanglement re-directs evolutionary pressure by selecting against high-frequency mutations that inactivate toxin function, which allows for the concomitant optimization of strain fitness and circuit stability during the course of an evolutionary stability assay. This pairing of gene entanglement with adaptive evolution yielded a kill-switch circuit in which all lineages remained functionally stable for >130 generations, which compares favorably with the most stable kill-switch circuits developed to date (79). As such, we posit that an adaptive laboratory evolution campaign following sequence entanglement can be an effective strategy for improving the fitness and stability of synthetic genetic circuits in an unbiased fashion, thereby obviating the need for manual circuit optimization efforts (20). A potential caveat, however, is that the mechanism of stabilization for this circuit involved a signification reduction in the expression of the gene of interest *relE*, which may not be a desired outcome for all genetic circuits or applications.

The concept of linking a burdensome GOI with an essential function to enhance mutational robustness has been previously implemented using non-entanglement-based methods such as transcriptionally linking an antibiotic resistance gene to circuit expression (31), using bi-directional promoters to control expression of both an essential gene and a GOI (30) and through synthetic addiction using ligand-responsive molecular biosensors that link production of a target metabolite to expression of an essential function (25). While the first two techniques enhanced the evolutionary stability of a circuit to varying degrees, these strategies remain prone to circuit inactivation by large insertions and deletions. Moreover, although synthetic addiction achieved exemplary stability, the availability of biosensor modules limits the broad utility of this strategy. Here we show that sequence entanglement is an alternative and effective strategy for protecting synthetic circuits by directly linking the sequence that confers the desired circuit function to cell viability. Importantly, sequence entanglement does not introduce extra circuit components to the system, minimizing the number of potential breakage points.

An important design consideration for imparting mutational robustness is the choice of the essential gene. Here, *ilvA* was made conditionally essential by knocking out the two annotated paths for isoleucine biosynthesis in *P. protegens* (Δ*ilvA*Δ*tdcB*). One potential limitation of this system could be the accumulation of compensatory mutations that abolish the necessity of *ilvA*. A recent study showed that *E. coli* acquires mutations to circumvent isoleucine auxotrophy at a high frequency in a Δ*ilvA*Δ*tdcB* strain via activation of an underground isoleucine biosynthesis pathway (through *metABC*) (80). Importantly, the *metABC* genes are not present in *P. protegens* and our Δ*ilvA*Δ*tdcB* strain exhibits a low escape ratio in minimal medium (Supplementary Figure S12; ∼10^−9^), indicating that this host cannot easily circumvent isoleucine auxotrophy.

Cross-feeding by non-deficient neighbors is another potential vulnerability. Studies in *E. coli* have shown that an isoleucine auxotroph can be stably maintained in a population containing isoleucine-producing variants (80–82). When propagating the *ilvA/relE*^RBS3^ circuit in the absence of isoleucine during the evolutionary stability assay, the escape ratio increased over the first 20 generations and then reached a plateau (Figure 4A). We speculate that this initial increase in escape ratio may be explained by cross-feeding activity that stabilized a subset of the population with inactivated *ilvA*/*relE* (Figure 4A). The ensuing dip in the escape ratio in several lineages suggests a shift in the composition back toward variants with a functional *relE*. One possibility is that the populations were overtaken by a mutation that renders lower *ilvA*/*relE* expression (e.g. mutations within *rhaRS*), which increases fitness by lessening *relE* toxicity but lowers isoleucine production such that the threshold amount of isoleucine needed to support an *ilvA* null mutant via cross-feeding is no longer reached. Indeed, population modeling predicts a crash in population size when the isoleucine concentration dips below a critical size (81). As such, non-isoleucine-producing cheaters probably cannot completely thwart the protective effects of entanglement but may impact population dynamics. Ultimately, the choice of an (conditionally) essential gene for entanglement should not only consider the flexibility and mutational protectiveness, but also be informed by knowledge of the target environment and host-specific modes of mutational escape.

Our results also highlight the importance of characterizing essentiality at the protein domain level when designing gene entanglements. While essential for growth in *E. coli* (35), the C-terminal regulatory domain of IlvA was found to be non-essential in *P. protegens* (Supplementary Figure S7A). This is consistent with the observation that frameshifts and deletions within the entangled region were a source of entanglement escape (Figures 3C and 5A). Interestingly, however, deletion of this domain reduced cell fitness, an effect that was particularly pronounced under conditions of low *ilvA* expression (Supplementary Figure S7B). It is thus tempting to speculate that as mutations accumulated in the *rhaR*/*rhaS* regulators and reduced *ilvA*/*relE* expression during the long-term evolutionary stability assay (Figure 4A), there may have been increased selective pressure to maintain the IlvA C-terminal regulatory domain, which in turn may have contributed to the stabilization of entangled *relE* (78). Altogether, these results suggest that more sequence-protective entanglement designs can probably be achieved by targeting regions within or in proximity to a more critical protein domain (e.g. enzyme active sites).

Overall, this study identified key strengths and limitations of the *ilvA*/*relE* entanglement pair that provide important design considerations for the utility of sequence entanglement. Ultimately, *ilvA/relE* represents a successful showcase of entanglement as a strategy to protect and improve the genetic stability of synthetic circuits, which may be useful to a wide variety of bioengineering applications ranging from the biocontainment of genetically engineered microorganisms to the stabilization of industrial protein expression systems. This may be especially relevant for current biocontainment strategies such as kill-switches, auxotrophies, codon recoding and others that are vulnerable to genetic mutations (17–19,69,83). Our results also highlight the generalizability of entanglement to other organisms, as the *ilvA/relE* gene pair was portable to an evolutionarily distant *Pseudomonas* species. Future studies will focus on further testing the generalizability of these results using additional entanglement pairs and by targeting entanglement locations within essential protein-coding domains.

## Supplementary Material

gkad484_Supplemental_File

## Data Availability

All relevant data are either within the manuscript or supplemental information files or have been deposited in public databases as listed in the Materials and Methods. The original *i**lvA/relE* vector along with the vectors containing the RBS3 modification and vectors from CB 2, CB 3 and CB 4 have been deposited in Addgene (plasmid number 201531–201535). All sequencing libraries are available at BioProject PRJNA970322 (https://www.ncbi.nlm.nih.gov/bioproject).
