# Supplementary material for: Prolonging genetic circuit stability through adaptive evolution of overlapping genes
Source: Nucleic Acids Res. 2023 Jun 1;51(13):7094–108. doi: 10.1093/nar/gkad484 (PMC10359631; doi:10.1093/nar/gkad484)
Supplement: gkad484_Supplemental_File [file gkad484_supplemental_file.pdf]

Supplemental Information for:

Prolonging Genetic Circuit Stability through Adaptive Evolution of Overlapping Genes

Jennifer L. Chlebek[1], Sean P. Leonard[1], Christina Kang-Yun[1], Mimi C. Yung[1], Dante P. Ricci[1], Yongqin Jiao[1], and Dan M. Park[1]*

[1]Biosciences and Biotechnology Division, Lawrence Livermore National Laboratory, Livermore, CA 94550

*Correspondence to: park36@llnl.gov

This document includes:

Supplemental Discussion

Figs. S1-S12

Tables S1-S4

Supplemental Reference

SUPPLEMENTAL DISCUSSION

Based on our observations in this work, we anticipate that entanglement designs will, in most cases, require post-entanglement optimization of an internal RBS to facilitate translation of the internal gene. In this proof-of-concept example, we achieved toxic levels of RelE using a *post-hoc* RBS optimization approach whereby strong RBS sites that minimize disruptive amino acid substitutions within IlvA were identified and introduced. Fortuitously, one such RBS site (RBS3) not only improved translation from the embedded *relE* gene but also enhanced the functionality of IlvA (**Fig. 2A-B** and **S3**). We posit that the entanglement of WT *relE* within the genetic region encoding the C-terminal regulatory domain of *ilvA* reduces or alters the autoregulation of IlvA; this phenomenon may also explain the diminished functionality of *ilvA*/*relE*^{STOP} compared to *ilvA*^{WT} in minimal medium without isoleucine (**Fig. 1A** and **2A**). Since the RBS modifications occur in the alpha-helical "neck" region that separates the catalytic and regulatory domains of IlvA (**Fig. 1A**), we speculate that the amino acid changes imposed by RBS3 (**Fig. S5,** *ilvA*$^{L333V/G334L}$) may have locked the N-terminus of IlvA into a catalytically active state. This would allow for the constitutive production of isoleucine and support the enhanced growth that we observed with the RBS3 modification without altering IlvA expression levels (**Fig. S6B**). Confirming this hypothesis will require further biochemical and structural characterization. While we do not expect this fortuitous result to generalize to other entanglement pairs, it does highlight an important design constraint of entanglement: mutations that improve the strength of the internal RBS may impact the fitness of the external entangled gene. Entanglement algorithms that incorporate RBS modifications along with fitness prediction would be highly valuable for future entanglement designs.

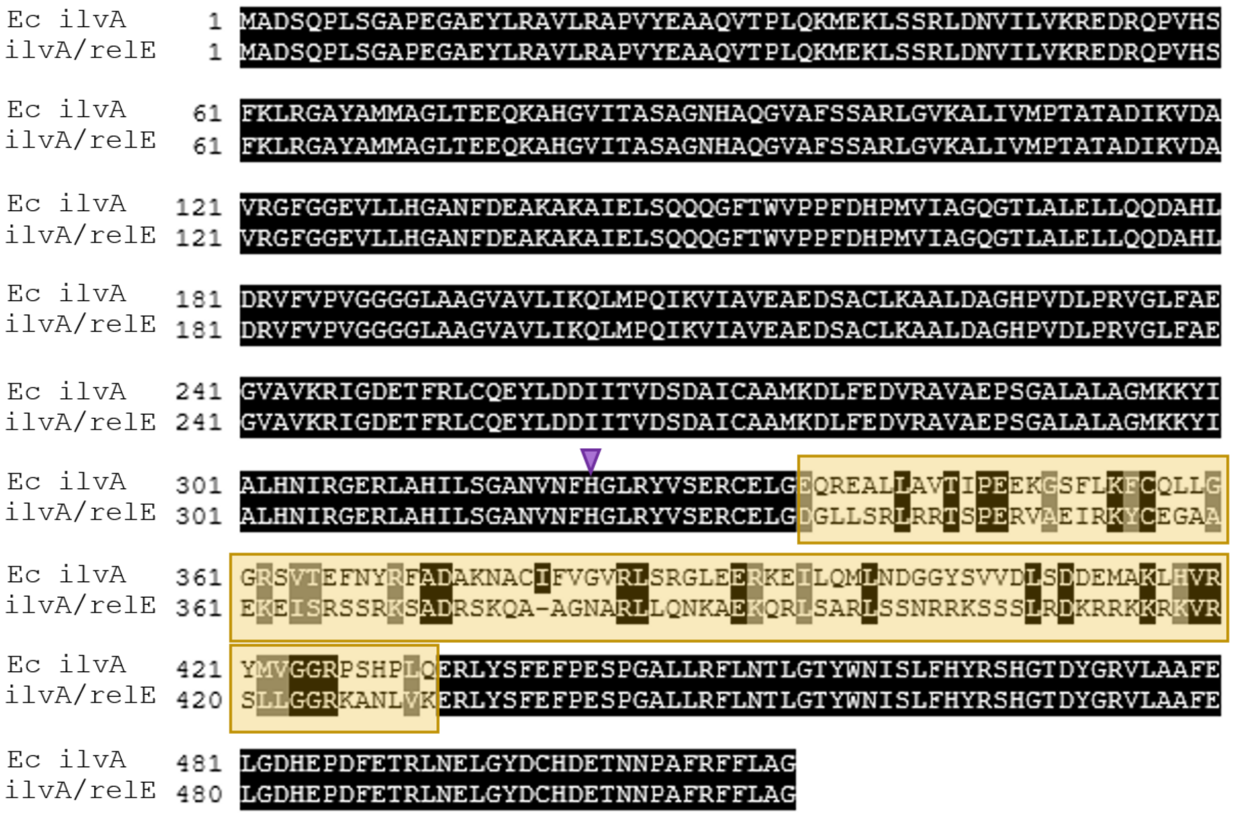

Fig S1. Amino acid sequence of *ilvA*/*relE* entanglement. Alignment of the amino acid sequence between *E. coli* $ilvA^{WT}$ and the *ilvA*/*relE* entanglement design. The orange box denotes the entanglement position. The purple triangle indicates the position of the C-terminal truncation ($ilvA^{\Delta H322\text{-}G514}$) seen in **Fig. S8**.

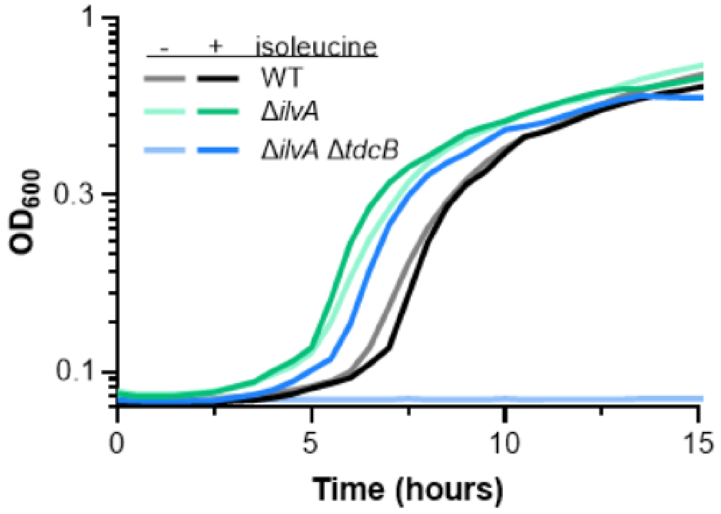

Fig S2. *ilvA* and *tdcB* are required for isoleucine biosynthesis in *P. protegens* Pf-5. In *P. protegens* Pf-5, *ilvA* and *tdcB*, a close homolog of *ilvA*, are both required for isoleucine biosynthesis. Strains were grown in minimal medium with or without the addition of isoleucine. Growth is reported as OD_{600} over time. Data are shown as the mean of 3 independent replicates.

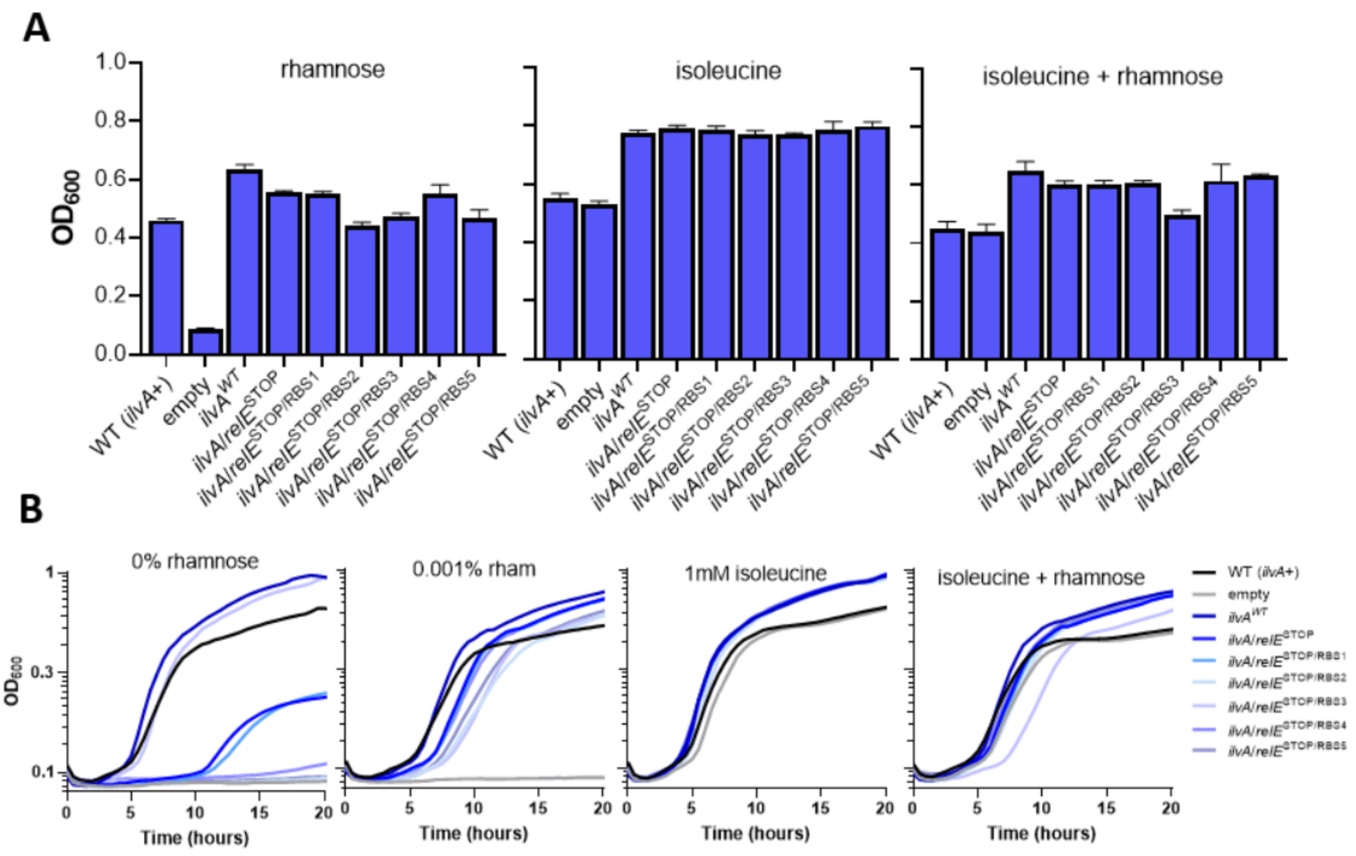

Fig S3. Testing the ability of *ilvA*/*relE*^{STOP} alleles to rescue isoleucine auxotrophy. Final density of isoleucine auxotroph (Δ*ilvA*Δ*tdcB)* strains with chromosomally integrated antitoxin (P_{cymR}-*relB*) harboring plasmids carrying the different alleles listed driven under the P_{rhaBAD} promoter. **(A)** Strains harboring *ilvA*/*relE*^{STOP} alleles containing different strength RBSs were grown in minimal medium with addition of either rhamnose (to induce the P_{rhaBAD} -*ilvA*/*relE* alleles*)* or isoleucine. Growth is reported as OD_{600} after 15 h. **(B)** Growth curves from data represented in figure **2A** and **S3A** in which growth is reported as OD_{600} over time. All data in bar graphs are shown as the mean ± SD and data in growth curves are shown as the mean. All data are representative of 3 independent replicates.

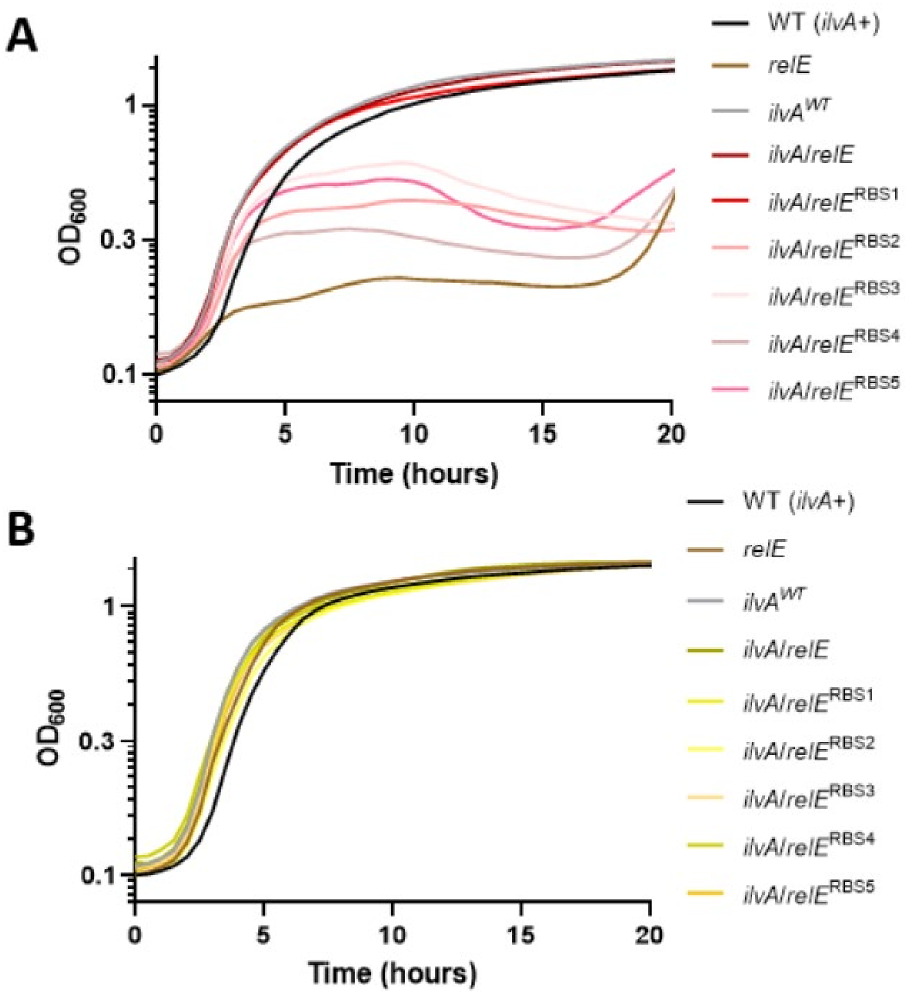

Fig S4. Testing capability of *relE* in *ilvA*/*relE* alleles to suppress growth. Growth curves represent the data from **Fig. 2B** in which growth is reported as OD_{600} over time. **(A)** Strains (Δ*ilvA*Δ*tdcB,* P_{cymR}-*relB*) harboring P_{rhaBAD}-*ilvA*/*relE* alleles with different strength RBSs were grown in rich medium to isolate *relE* toxicity. Experiments were performed without rhamnose induction of *ilvA*/*relE* expression. **(B)** To rescue growth, the antitoxin was induced by addition of cumate. Data is shown as the mean of 3 independent replicates.

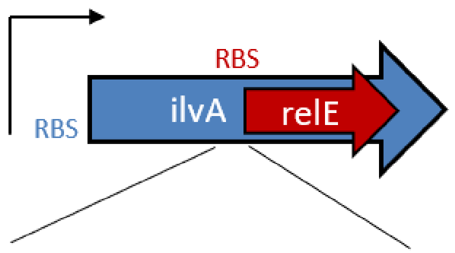

Predicted translation rate

RBS^WT GCTACGTCTCAGAACGCTGCGAACTGGGCG - 13.6
LRYVSERCELG

RBS1 GCTACGTCTCAGAACGgTGCGAAggGGGCG – 127.6
LRYVSERCE**G**G

RBS2 GCTACGTCTCAGAAaGggGgGAgtTaGGCG – 506.49
LRYVSER**G**ELG

RBS3 GCTACGTCTCAGAAaGaTGtGAggTGttaG - 1036
LRYVSERCE**VL**

RBS4 GCTACGTCTCAGAAaaaTGtGAggTGttaG - 4787
LRYVSE**Y**CE**VL**

RBS5 GCTACGTCTCAGAAaGggGgGAggTGttaG – 12,999
LRYVSER**GEVL**

Fig S5. Optimization of internal RBS to improve RelE expression. The nucleotide sequence (top) and amino acid sequence (bottom) of the internal RBS region within *ilvA* (red underline), just upstream of the start codon of entangled *relE,* is listed next to each variant tested. Changes to the nucleotide sequence of *ilvA* are seen with lowercase letters and the corresponding changes in the amino acid sequence are depicted in bold red font. The predicted translation rate was determined using the Salis Lab RBS calculator (1). The rates are given on a proportional scale ranging from 1 to 100,000+.

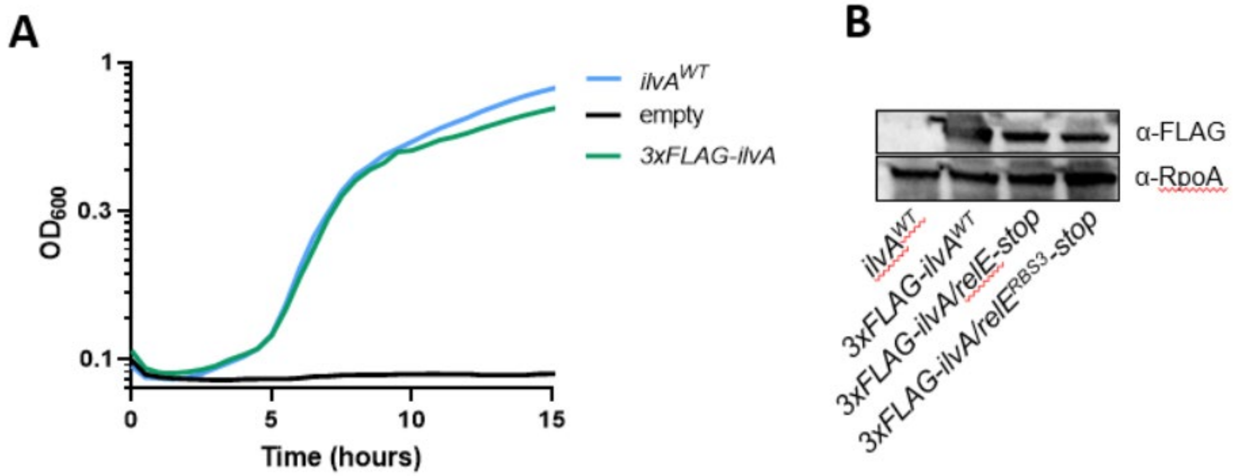

Fig S6. Improved RBS modification does not affect threonine deaminase expression. (A) Growth curve in minimal medium without isoleucine shows that 3xFLAG tagged *ilvA* constructs are functional for isoleucine biosynthesis. Growth is reported as OD_{600} over time. **(B)** Western blot testing whether entanglement and/or RBS3 modification affects expression of threonine deaminase. Strains were grown overnight with rhamnose for induction of the *ilvA* variants. Growth curve data are shown as the mean and all data are representative of 3 independent replicates.

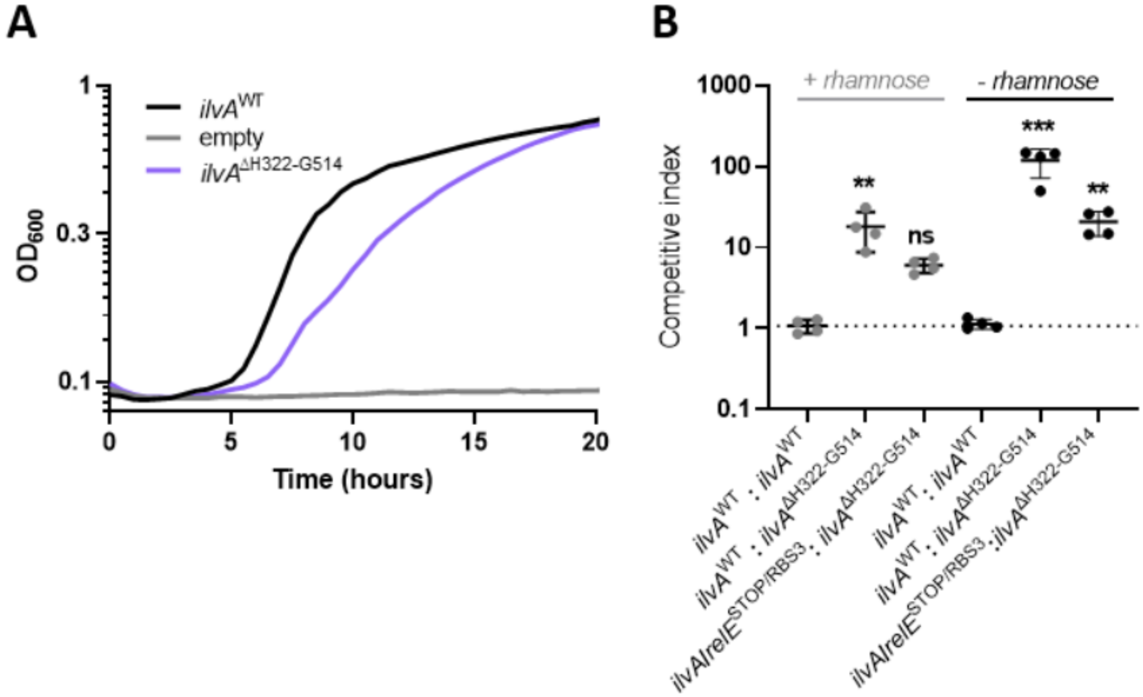

Fig S7. The C-terminus of *ilvA* is important for maximal cell fitness. (A) A Δ*ilvA*Δ*tdcB* P_{cymR}-*relB P. protegens* strain harboring a vector containing the listed alleles under a rhamnose inducible promoter were grown in minimal medium without isoleucine and with rhamnose to probe the function of *ilvA*. The *ilvA*$^{\Delta H322\text{-}G514}$ allele contains a C-terminal truncation of *ilvA* immediately upstream of the internal RBS for *relE*. Growth is reported as OD_{600} over time. **(B)** Competition assay in which *ilvA*^{WT}, *ilvA*/*relE*^{RBS3} and *ilvA*$^{\Delta H322\text{-}G514}$ were grown in a 1:1 co-culture in minimal medium in the presence or absence of rhamnose (0.001%). Strains were differentially marked with chromosomally integrated antibiotic cassettes, tetracycline and gentamicin. The competitive index was calculated as the CFU ratio of mutant/parent after growth for 48 hours divided by the CFU ratio of the mutant/parent in the initial inoculum. Growth curve data for panel **A** are shown as the mean of at 3 independent replicates and data for panel **B** are shown as the mean ± SD of 4 independent replicates. Asterisk(s) directly above data denote comparisons to the *ilvA*^{WT}: *ilvA*^{WT} co-culture for each condition. Comparisons were made by one-way ANOVA with Dunnet's post test. *** = P < 0.001, ** = P < 0.01, ns = not significant.

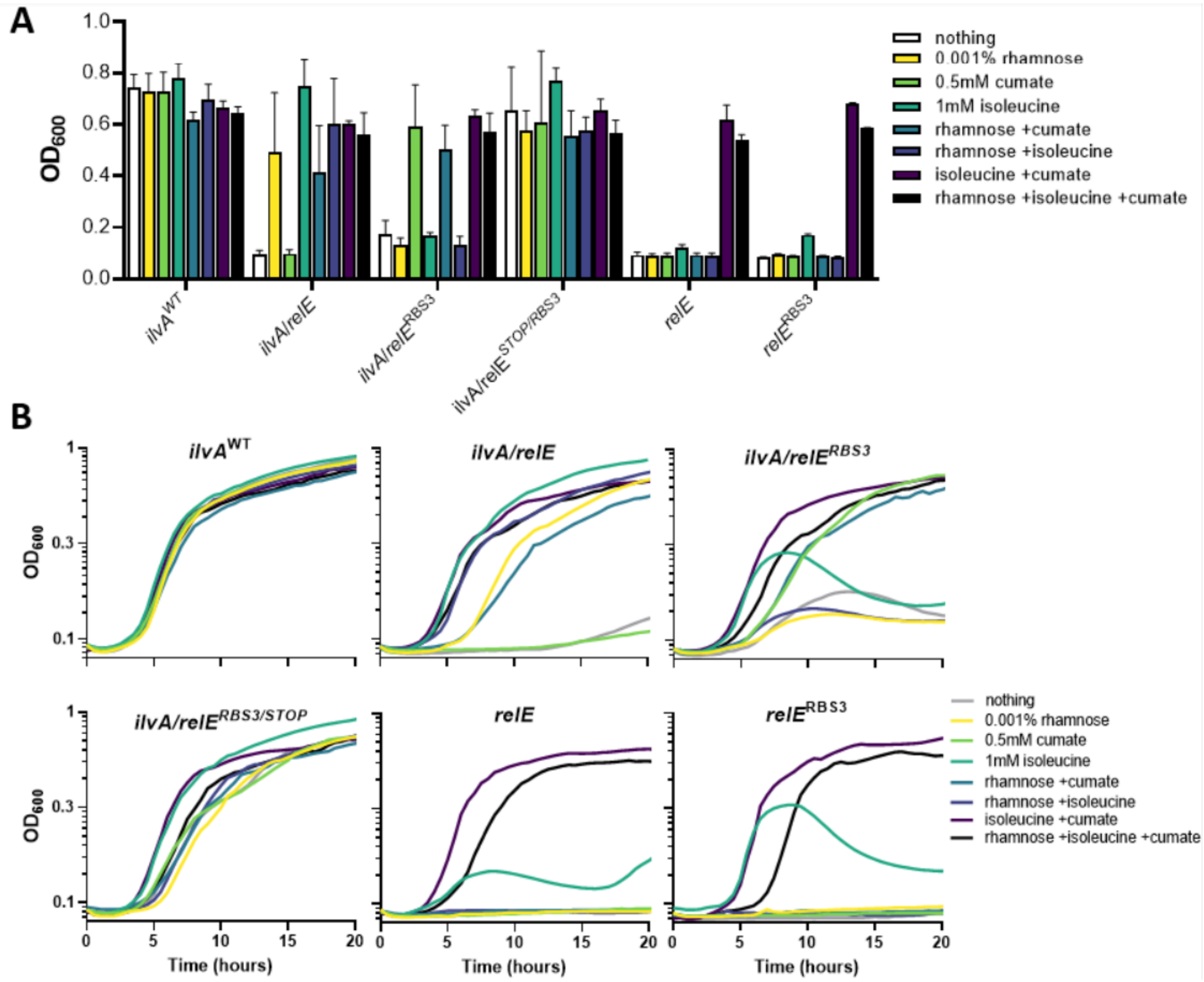

Fig S8. The *ilvA*/*relE*^{RBS3} and *relE*^{RBS3} alleles impart a growth defect in the absence of the antitoxin. Final density of isoleucine auxotroph (Δ*ilvA*Δ*tdcB)* strains with chromosomally integrated antitoxin (P_{cymR}-*relB*) and harboring plasmids carrying the different alleles listed under the P_{rhaBAD} promoter. **(A)** Strains harboring *ilvA*/*relE*^{STOP} alleles containing different strength RBSs were grown in minimal medium with addition of inducers as listed. Growth is reported as OD_{600} after 15 h. **(B)** Growth curves from data in panel **A** in which the OD_{600} was measured over time for each strain. Data in bar graphs are shown as the mean ± SD and data in growth curves are shown as the mean. All data are representative of 3 independent replicates.

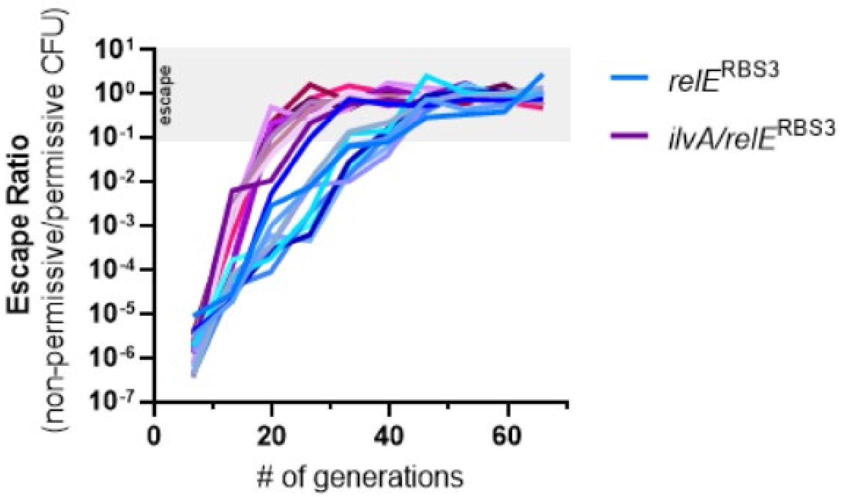

Fig S9. Evolutionary stability of non-entangled *relE*$^{\mathbf{RBS3}}$. Independent lineages (Δ*ilvA*Δ*tdcB* P$_{cymR}$-*relB P. protegens*) harboring *relE*^{RBS3} (blue lines) were grown in minimal medium with rhamnose (to induce *relE*^{RBS3}) and cumate (to induce antitoxin) in the presence of isoleucine. Each day (~6.6 generations) the cultures were diluted 1:1,000 in fresh medium and plated for CFU on toxin permissive and non-permissive conditions and a survival frequency was calculated. The grey bar indicates toxin escape ratio ≥ 10^{-1} (10%). Each line represents data from one of ten independent replicates picked from single colonies. The RBS3 modification was engineered to drive the expression of P$_{rham}$-*relE* to a similar level as *ilvA*/*relE*^{RBS3} (**Fig. S8**). Escape ratio data for the *ilvA/relE*^{RBS3} strain grown with isoleucine (purple lines) are identical to those shown in **Fig. 4A** and are plotted again here to facilitate comparisons to the *relE*^{RBS3} strain.

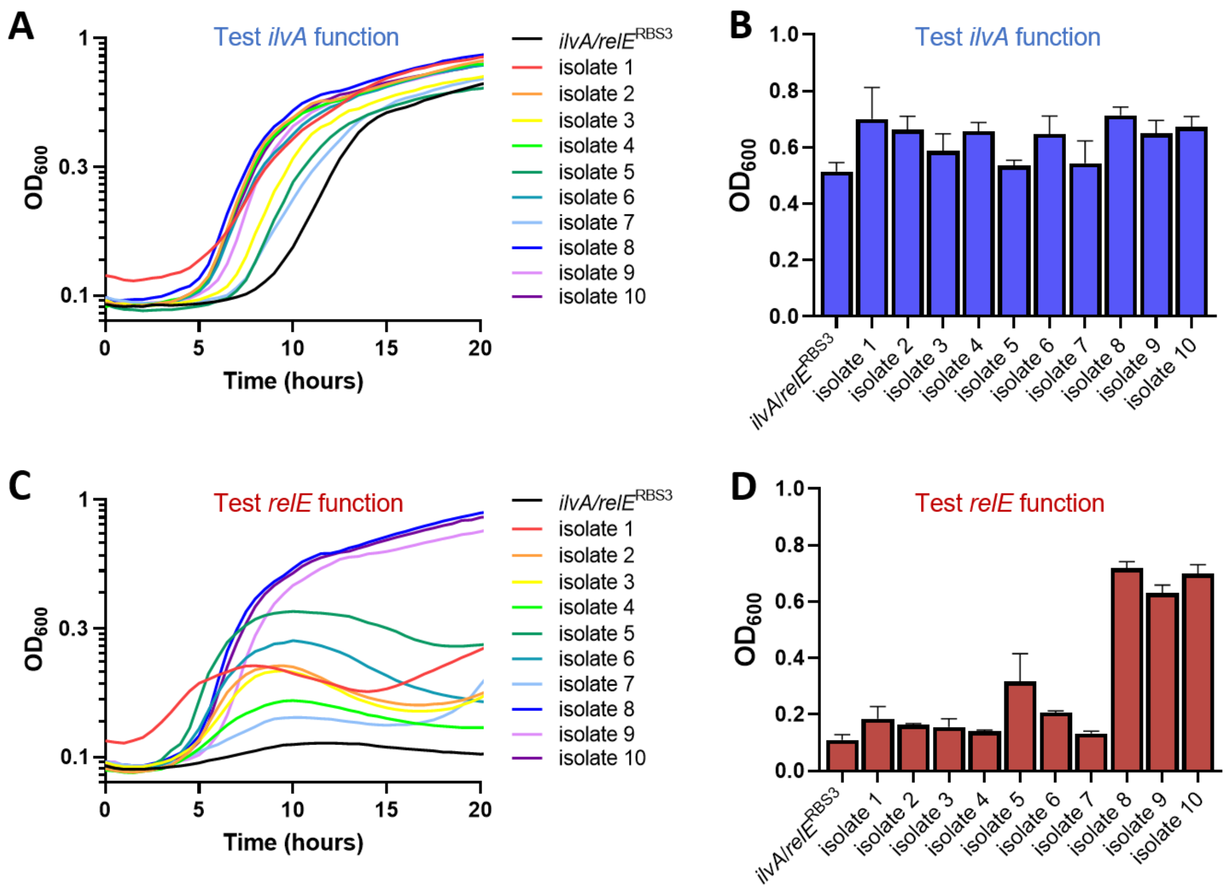

Fig S10. Growth of isolated colonies from each lineage of the long-term evolutionary stability assay. Single colonies were isolated from each lineage grown without isoleucine on permissive medium during the final passage of the long-term evolutionary stability assay. Each original colony was grown in minimal medium and the OD_{600} was measured over time (panels **A** and **C**) and a bar graph representing OD_{600} after 15 h of growth was generated (panels **B** and **D**). **(A**, **B)** To assess isoleucine auxotrophy, minimal medium without isoleucine was supplemented with rhamnose and cumate. **(C, D)** To assess *relE* toxicity, minimal medium was supplemented with rhamnose and isoleucine and without cumate. Data in bar graphs are shown as the mean ± SD and data in growth curves are shown as the mean. All data are representative of 3 independent replicates.

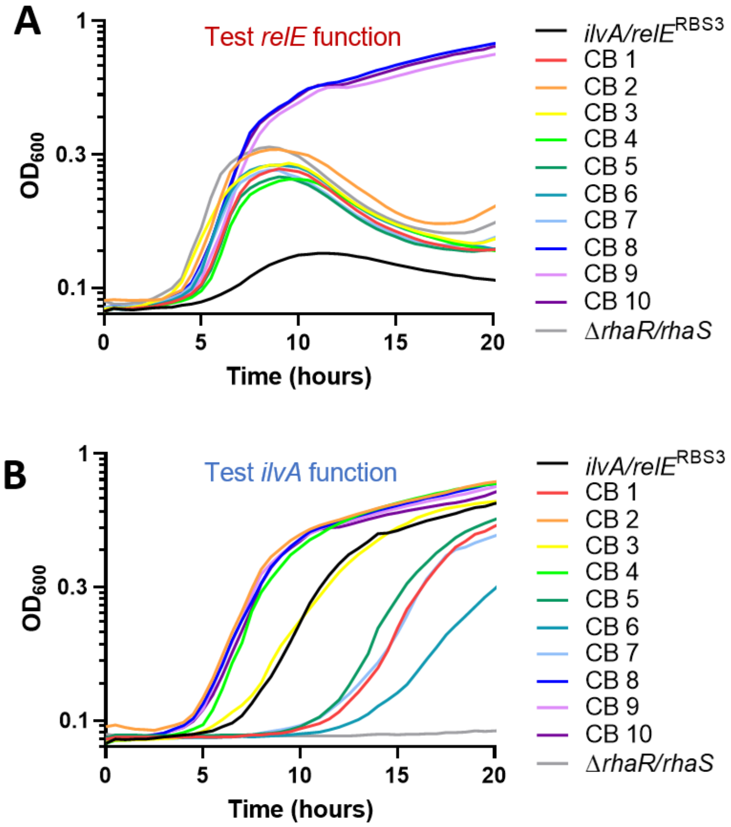

Fig S11. Growth of new clean background (CB) strains harboring isolated vectors from each lineage of the long-term evolutionary stability assay. Vectors were isolated from the colonies of each lineage grown without isoleucine and transformed into a clean genetic background (Δ*ilvA*Δ*tdcB* P_{cymR}-*relB)* and were grown in **(A)** minimal medium with isoleucine and rhamnose and without cumate to test *relE* activity or **(B)** in minimal medium without isoleucine with addition of rhamnose and cumate to probe *ilvA* function. Growth curves represent data from figure **5B** and **5C**. Data are shown as the mean of 3 independent replicates.

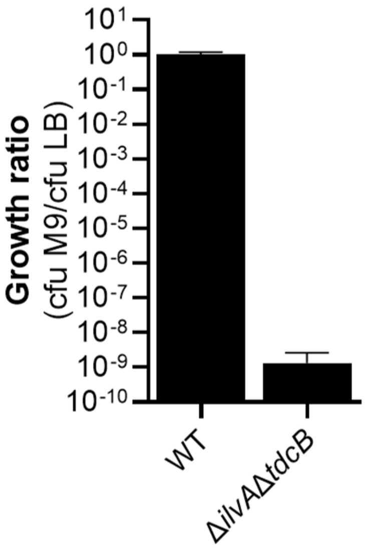

Fig S12. Growth ratio of cells that can survive on minimal medium. The indicated strains were grown overnight in LB medium, washed twice in minimal medium and then serially diluted onto either LB-agar or M9-agar plates without isoleucine. After 48 h of growth, growth ratio was calculated by taking the CFU/mL on M9 plates divided by the CFU/mL on LB plates. No colonies were observed for one replicate for the Δ*ilvA*Δ*tdcB* strain and this was reported as a limit of detection (one colony) at the lowest dilution plated. Data are shown as the mean ± SD of 4 independent replicates.

Table S1. Primers used in this study

Primer name	Sequence (5' –> 3')	Description
Phram_for_ilvA_F	cttctgactgagttgcacgctg	amplify pJUMP24-T24 vector for insert downstream of P_{rhaBAD} F
Phram_for_ilvA_R	ctccttcttaaagttaaacaagtacgctagc	amplify pJUMP24-T24 vector for insert downstream of P_{rhaBAD} R
ilvA_for_pjump24_F	aactttaagaaggagatatacaatggctgactcgcaacccctg	amplify ilvA for insert into pJUMP24-T24-P_{rhaBAD} F
ilvA_for_pJUMP24_R	caactcagtcagaagctaacccgccaaaaagaacctgaacg	amplify ilvA for insert into pJUMP24-T24-P_{rhaBAD} R
tdcB_amp_F	aactttaagaaggagatatacaatgagtcgccccagccgttc	amplify tdcB for insert into pJUMP24-T24-P_{rhaBAD} F
tdcB_amp_R	caactcagtcagaagctacaaggcggcgttgctc	amplify tdcB for insert into pJUMP24-T24-P_{rhaBAD} R
ilvA_UR_F	tccgatcgagctcgagttgccgttgtcggtgatcac	amplify UP arm for ΔilvA F
ilvA_UR_R	acagctgcttgacgtactgttcgagcatcg	amplify UP arm for ΔilvA R
ilvA_DR_F	acgtcaagcagctgttcctcggctgaacg	amplify DOWN arm for ΔilvA F
ilvA_DR_R	tgtacagggccctagccaggcaatcgagatccagct	amplify DOWN arm for ΔilvA R
tdcB_UR_F	tccgatcgagctcgagttgtactggcccttgctgg	amplify UP arm for ΔtdcB F
tdcB_UR_R	tgctcaggctggggcgactcatggtg	amplify UP arm for ΔtdcB R
tdcB_DR_F	gccccagcctgagcaacgccgccttg	amplify DOWN arm for ΔtdcB F
tdcB_DR_R	tgtacagggccctagggtgaccggtgacggcttc	amplify DOWN arm for ΔtdcB R
pRE118_amp_F	gagaagcggtgtaagtgaactgc	amplify prE118 for insert ΔilvA or ΔtdcB sequence F
pRE118_amp_R	gccatgaatggcgttggatgc	amplify prE118 for insert ΔilvA or ΔtdcB sequence R
relB_amp_for_cymR_F	gagaaattaaccatgggtagcattaacctgcgtattgac	amplify relB for insert into pUC18_miniTn7_CymR_P_{cymR} F
relB_amp_for_cymR_R	aggaattcctgcagctcagagttcatccagcgtcacac	amplify relB for insert into pUC18_miniTn7_CymR_P_{cymR} R
Tn7_amp_for_relB_F	gctgcaggaattcctcgagaagc	amplify pUC18_miniTn7_CymR_P_{cymR} vector for insert F
CymR_for_dcas9_R	catggttaatttctcctctttaattctag	amplify pUC18_miniTn7_CymR_P_{cymR} vector for insert R
relE_RBS1_F	cggtgcgaagggggcgatggcctactttctagacttcg	insert RBS1 in front of relE in ilvA/relE F
relE_RBS1_R	gccccccttcgcaccgttctgagacgtagcgcaggc	insert RBS1 in front of relE in ilvA/relE R
relE_RBS2_F	agatgtgaggtgttagatggcctactttctagacttcgacg	insert RBS3 in front of relE in ilvA/relE F

relE_RBS2_R	taacacctcacatctttctgagacgtagcgcaggc	insert RBS3 in front of relE in ilvA/relE R
relE_RBS3_F	aaatgtgaggtgttagatggcctactttctagacttcgacg	insert RBS4 in front of relE in ilvA/relE F
relE_RBS3_R	taacacctcacatttttctgagacgtagcgcaggc	insert RBS4 in front of relE in ilvA/relE R
relE_RBS5_F	agggggggagttaggcgatggcctactttctagacttcg	insert RBS2 in front of relE in ilvA/relE F
relE_RBS5_R	gcctaactccccccctttctgagacgtagcgcaggc	insert RBS2 in front of relE in ilvA/relE R
relE_RBS6_F	agggggggaggtgttagatggcctactttctagacttcgacg	insert RBS5 in front of relE in ilvA/relE F
relE_RBS6_R	taacacctccccccctttctgagacgtagcgcaggc	insert RBS5 in front of relE in ilvA/relE R
pJUMP24_amp_for_term_F	ggagttttgcaggtgccttgg	amplify pJUMP24 vector for inserting T24 terminator F
pJUMP24_amp_for_term_R	gacaaatccgccgccctagac	amplify pJUMP24 vector for inserting T24 terminator R
T24_amp_F	cacctgcaaaactcctcgagaaagcaagctgataaacc g	amplify T24 terminator for insert into pJUMP24 vector F
T24_amp_R	ggcggcggatttgtctatagggcgaattggcgggagag	amplify T24 terminator for insert into pJUMP24 vector R
amp_for_rham_F	tcgagaaagcaagctcgtattacgcgcgctcactg	amplify pJUMP24-T24 for insert P_{rhaBAD} promoter F
amp_for_rham_R	acctgaatcgccagcctatagggcgaattggagctccac	amplify pJUMP24-T24 for insert P_{rhaBAD} promoter R
rham_for_gfp_F	cgcggccgcttctaggctggcgattcaggttcatcatg	amplify P_{rhaBAD} promoter for insert into pJUMP24-T24 F
rha_for_gfp_R	tagcactgtacctaggttcattacgaccagtctaaaaagcg	amplify P_{rhaBAD} promoter for insert into pJUMP24-T24 R
PJC010	aactttaagaaggagatatacaatggcctactttctagactt cgacgaac	amplify relE for insert into pJUMP24-T24-P_{rhaBAD} F
PJC011	caactcagtcagaagttacaagattcgctttacggcctcc	amplify relE for insert into pJUMP24-T24-P_{rhaBAD} R
PJC032	ctaacacctcacatctaaagttaaacaagtacgctagcac tgtac	RBS2 alteration for P_{rhaBAD}-relE R
PJC063	tccaccacttccacctgccattgtatatctccttcttaaagtta aacaagtacgc	amplify ilvA for insert Nterm 3xFLAG R
PJC064	gcaggtggagcaggtggagctgactcgcaacccctgtcc	amplify ilvA for insert Nterm 3xFLAG F
PJC070	gcaggtggaagtggtggagattataaagaccatgatggtg actacaaggatcacgacattgattataaggatgacgatga caaagcaggtggagcaggtgga	triple 3xFLAG internal sequence F
PJC071	tccacctgctccacctgctttgtcatcgtcatccttataatcaa tgtcgtgatccttgtagtcaccatcatggtctttataatctcca ccacttccacctgc	triple 3xFLAG internal sequence R
PJC121	cttctgactgagttgcacgctg	amplify pJUMP24-T24 vector for insert

		downstream of P_{rhaBAD} alternate F
PJC122	tgcataaaaactgttgtaattcattaagcattctgc	amplify pJUMP24-T24 vector for insert downstream of P_{rhaBAD} alternate R
PJC160	gaacttctaaggcctgcgctacgtc	insert stop codon for ilvA Δ322-514aa F
PJC161	cgcaggccttagaagttcacgttggc	insert stop codon for ilvA Δ322-514aa R
PJC226	cgttgctgctccataacatcaaac	amplify pUC18_miniTn7_CymR_P_{cymR}-*relB* to swap antibR R
PJC227	caattcgttcaagccgagatcg	amplify pUC18_miniTn7_CymR_P_{cymR}-*relB* to swap antibR F
PJC228	gtttgatgttatggagcagcaacgatgaaatctaacaatgc gctcatcg	amplify TetR for insert in miniTN7 vector F
PJC229	cgatctcggcttgaacgaattgtcaggtcgaggtggcccg	amplify TetR for insert in miniTN7 vector R
PJC234	aacagtttttatgcaactggcctcctgatgtcgtc	amplify pJUMP24-T24 to delete rhaR rhaS F
PJC122	tgcataaaaactgttgtaattcattaagcattctgc	amplify pJUMP24-T24 to delete rhaR rhaS R
PJC215	tactagtagcggccgctgcag	Amplify evolved ilvA/relE RBS3 vectors for insert gfp behind promoter F
PJC216	ctgcagcggccgctactagta	Amplify gfp for cloning into evolved vectors R
PJC201	cgctttttagactggtcgtaatgaacc	Amplify gfp for cloning into evolved vectors F
PJC235	tgtatatctccttcttaaagttaaacaagtacgc	Amplify evolved ilvA/relE RBS3 vectors for insert gfp behind promoter R

Table S2. ***Pseudomonas protegens*** **Pf-5 strains generated and used in this study**

Strain name in manuscript	Genotype of chromosome	Genotype of plasmid (all KanR)	Figure	Strain # (this study)
ilvA^{WT}	ΔilvA ΔtdcB PcymR-relB @glmS	pJUMP24-T24-P_{rhaBAD}-Ec ilvA	2A-B, S3, S4, S6, S6, S7, S8	SJC076
ilvA/relE^{STOP}	ΔilvA ΔtdcB PcymR-relB @glmS	pJUMP24-T24-PrhaBAD-ilvA/relE-STOP	2A, S3	SJC077
ilvA/relE$^{STOP/RBS1}$	ΔilvA ΔtdcB PcymR-relB @glmS	pJUMP24-T24-PrhaBAD-ilvA/relE-STOP RBS1	2A, S3	SJC078
ilvA/relE$^{STOP/RBS3}$	ΔilvA ΔtdcB PcymR-relB @glmS	pJUMP24-T24-PrhaBAD-ilvA/relE-STOP RBS3	2A, S3, S8	SJC079
ilvA/relE$^{STOP/RBS4}$	ΔilvA ΔtdcB PcymR-relB @glmS	pJUMP24-T24-PrhaBAD-ilvA/relE-STOP RBS4	2A, S3	SJC080

ilvA/*relE*$^{STOP/RBS2}$	ΔilvA ΔtdcB PcymR-relB @glmS	pJUMP24-T24-PrhaBAD-ilvA/relE-STOP RBS2	2A, S3	SJC081
ilvA/*relE*$^{STOP/RBS5}$	ΔilvA ΔtdcB PcymR-relB @glmS	pJUMP24-T24-PrhaBAD-ilvA/relE-STOP RBS5	2A, S3	SJC082
empty	ΔilvA ΔtdcB PcymR-relB @glmS	pJUMP24-T24-PrhaBAD-gfp	2A-B, S3, S4, S6, S7	SJC094
WT (*ilvA*+)	PcymR-relB @glmS	pJUMP24-T24-PrhaBAD-gfp	2A-B, S3, S4, S6	SJC347
ilvA/*relE*	ΔilvA ΔtdcB PcymR-relB @glmS	pJUMP24-T24-PrhaBAD-ilvA/relE	2B, S4, S8	SJC070
ilvA/*relE*^{RBS1}	ΔilvA ΔtdcB PcymR-relB @glmS	pJUMP24-T24-PrhaBAD-ilvA/relE RBS1	2B, S4	SJC071
ilvA/*relE*^{RBS3}	ΔilvA ΔtdcB PcymR-relB @glmS	pJUMP24-T24-PrhaBAD-ilvA/relE RBS3	2B, 3, 4, 5, S4, S6, S8, S10,	SJC072
ilvA/*relE*^{RBS4}	ΔilvA ΔtdcB PcymR-relB @glmS	pJUMP24-T24-PrhaBAD-ilvA/relE RBS4	2B, S4	SJC073
ilvA/*relE*^{RBS2}	ΔilvA ΔtdcB PcymR-relB @glmS	pJUMP24-T24-PrhaBAD-ilvA/relE RBS2	2B, S4	SJC074
ilvA/*relE*^{RBS5}	ΔilvA ΔtdcB PcymR-relB @glmS	pJUMP24-T24-PrhaBAD-ilvA/relE RBS5	2B, S4	SJC075
relE	ΔilvA ΔtdcB PcymR-relB @glmS	pJUMP24-T24-PrhaBAD-relE	2B, S4, S8	SJC175
relE^{RBS3}	ΔilvA ΔtdcB PcymR-relB @glmS	pJUMP24-T24-PrhaBAD-relE RBS3	4B, S8	SJC212
CB 3	ΔilvA ΔtdcB PcymR-relB @glmS	PrhaBAD-ilvA/relE RBS3 from isolate 3	5B-C, S11	SJC427
CB 4	ΔilvA ΔtdcB PcymR-relB @glmS	PrhaBAD-ilvA/relE RBS3 from isolate 4	5B-C, S11	SJC428
CB 2	ΔilvA ΔtdcB PcymR-relB @glmS	PrhaBAD-ilvA/relE RBS3 from isolate 2	5B-C, 6C, S11	SJC429
CB 1	ΔilvA ΔtdcB PcymR-relB @glmS	PrhaBAD-ilvA/relE RBS3 from isolate 1	5B-C, S11	SJC430
CB 5	ΔilvA ΔtdcB PcymR-relB @glmS	PrhaBAD-ilvA/relE RBS3 from isolate 5	5B-C, S11	SJC431
CB 6	ΔilvA ΔtdcB PcymR-relB @glmS	PrhaBAD-ilvA/relE RBS3 from isolate 6	5B-C, S11	SJC432
CB 7	ΔilvA ΔtdcB PcymR-relB @glmS	PrhaBAD-ilvA/relE RBS3 from isolate 7	5B-C, S11	SJC433
CB 9	ΔilvA ΔtdcB PcymR-relB @glmS	PrhaBAD-ilvA/relE RBS3 from isolate 9	5B-C, S11	SJC434
CB 8	ΔilvA ΔtdcB PcymR-relB @glmS	PrhaBAD-ilvA/relE RBS3 from isolate 8	5B-C, S11	SJC435
CB 10	ΔilvA ΔtdcB PcymR-relB @glmS	PrhaBAD-ilvA/relE RBS3 from isolate 10	5B-C, S11	SJC436
Δ*rhaR*Δ*rhaS*	ΔilvA ΔtdcB PcymR-relB @glmS	pJump24-T24-PrhaBAD-ilvA/relE RBS3 ΔrhaRΔrhaS	5B, S9, S11	SJC449

parent (gentR)	ΔilvA ΔtdcB PcymR-relB @glmS GentR	pJUMP24-T24-PrhaBAD-ilvA/relE RBS3	6B	SJC451
parent (tetR)	ΔilvA ΔtdcB PcymR-relB @glmS TetR	pJUMP24-T24-PrhaBAD-ilvA/relE RBS3	6B	SJC457
CB 3	ΔilvA ΔtdcB PcymR-relB @glmS TetR	PrhaBAD-ilvA/relE RBS3 from isolate 3	6B	SJC458
CB 4	ΔilvA ΔtdcB PcymR-relB @glmS TetR	PrhaBAD-ilvA/relE RBS3 from isolate 4	6B	SJC459
CB 2	ΔilvA ΔtdcB PcymR-relB @glmS TetR	PrhaBAD-ilvA/relE RBS3 from isolate 2	6B	SJC460
parent vector		pJUMP24-T24-PrhaBAD-gfp	6B	SJC412
empty		pJUMP24-T24-P_{rhaBAD}-Ec ilvA	6B	SJC413
vector 3		PrhaBAD-gfp from isolate 3	6B	SJC437
vector 4		PrhaBAD-gfp from isolate 4	6B	SJC438
vector 2		PrhaBAD-gfp from isolate 2	6B	SJC439
vector 1		PrhaBAD-gfp from isolate 1	6B	SJC440
vector 5		PrhaBAD-gfp from isolate 5	6B	SJC441
vector 6		PrhaBAD-gfp from isolate 6	6B	SJC442
vector 7		PrhaBAD-gfp from isolate 7	6B	SJC443
ΔrhaRΔrhaS		pJump24-T24-PrhaBAD-gfp ΔrhaRΔrhaS	6B	SJC444
WT			S1, S12	DP1345
ΔilvA	ΔilvA		S1	DP1539
ΔilvA ΔtdcB	ΔilvA ΔtdcB		S1, S12	DP1545
3xFLAG-ilvA	ΔilvA ΔtdcB PcymR-relB @glmS	pJUMP24-T24-PrhaBAD-3xFLAG-Ec ilvA	S6	SJC327
ilvA$^{\Delta 322\text{-}514aa}$	ΔilvA ΔtdcB	pJUMP24-T24-P_{rhaBAD}-Ec ilvA Δ322-514aa	S7	SJC275
isolate 3	ΔilvA ΔtdcB PcymR-relB @glmS	pJUMP24-T24-PrhaBAD-ilvA/relE RBS3 from D20-1	5A, S10	SJC464
isolate 4	ΔilvA ΔtdcB PcymR-relB @glmS	pJUMP24-T24-PrhaBAD-ilvA/relE RBS3 from D20-2	5A, S10	SJC465
isolate 2	ΔilvA ΔtdcB PcymR-relB @glmS	pJUMP24-T24-PrhaBAD-ilvA/relE RBS3 from D20-3	5A, S10	SJC466
isolate 1	ΔilvA ΔtdcB PcymR-relB @glmS, fliA^{V220G}, A→G @ 1,270,183 nt	pJUMP24-T24-PrhaBAD-ilvA/relE RBS3 from D20-4	5A, S10	SJC467
isolate 5	ΔilvA ΔtdcB PcymR-relB @glmS, uvrC^{P90R}	pJUMP24-T24-PrhaBAD-ilvA/relE RBS3 from D20-5	5A, S10	SJC468
isolate 6	ΔilvA ΔtdcB PcymR-relB @glmS, PFL_RS25775$^{\Delta 18bp}$	pJUMP24-T24-PrhaBAD-ilvA/relE RBS3 from D20-6	5A, S10	SJC469
isolate 7	ΔilvA ΔtdcB PcymR-relB @glmS	pJUMP24-T24-PrhaBAD-ilvA/relE RBS3 from D20-7	5A, S10	SJC470

isolate 9	ΔilvA ΔtdcB PcymR-relB @glmS, PFL_RS29355^{V88V}	pJUMP24-T24-PrhaBAD-ilvA/relE RBS3 from D20-8	5A, S10	SJC471
isolate 8	ΔilvA ΔtdcB PcymR-relB @glmS, (CGGTGA)3→4 @ 1,752,664 nt	pJUMP24-T24-PrhaBAD-ilvA/relE RBS3 from D20-9	5A, S10	SJC472
isolate 10	ΔilvA ΔtdcB PcymR-relB @glmS	pJUMP24-T24-PrhaBAD-ilvA/relE RBS3 from D20-10	5A, S10	SJC473

† Strain numbers refer to an internal strain tracking system at LLNL. Strain requests will be granted upon request of the authors.

Table S3. List of mutations in each sequenced strain of this study

Fig & replicate	Location	Position	Mutation	Annotation	Gene	Description
Fig 3C, yes, rep 1	chromo			cymR$^{V147H, \Delta 12bp}$	cymR	
Fig 3C, yes, rep 2	chromo			cymR$^{V147H, \Delta 12bp}$	cymR	
Fig 3C, yes, rep 3	chromo			cymR$^{V147H, \Delta 12bp}$	cymR	
Fig 3C, yes, rep 4	chromo			cymR^{A156P}	cymR	
Fig 3C, yes, rep 5	chromo			cymR$^{V147H, \Delta 12bp}$ & cymR$^{L180+1bp*}$	cymR	
Fig 3C, yes, rep 6	chromo			cymR^{T15I}	cymR	
Fig 3C, yes, rep 7	vector			relE$^{R81E\text{-}\Delta 1bp*}$, ilvA$^{K415R\text{-}\Delta 1bp*}$	[relE]–[ilvA]	
Fig 3C, yes, rep 8	vector			relE^{G46E}, ilvA$^{G419\text{-}\Delta 31bp*}$	[relE]–[ilvA]	
Fig 3C, No, rep 1	vector			ΔilvA^{150bp} - ilvA^{1470bp}	ilvA	
Fig 3C, No, rep 2	vector			ΔilvA^{608bp} - ilvA^{1473bp}	ilvA	
Fig 3C, No, rep 3	vector			ΔT24^{123bp} - ilvA^{302bp}	ilvA	
Fig 3C, No, rep 4	vector			ΔilvA^{883bp} - ilvA^{1051bp}/relE^{48bp}	[relE]–[ilvA]	
Fig 3C, No, rep 5	vector			ΔrhaR^{180bp} - ilvA^{233bp}	[rhaR]–[ilvA]	
Fig 3C, No, rep 6	vector			ΔoriV^{130bp} - ilvA^{95bp}	pRO1600 Rep ← / –	
Fig 3C, No, rep 7	vector			ΔrhaR^{8bp} - ilvA^{482bp}	[rhaR]–[ilvA]	
Fig 3C, No, rep 8	vector			ΔrhaR^{829bp} - ilvA^{1071bp}/relE^{68bp}	[rhaR]–[ilvA]	
Fig 5A, no ile, isolate 1	chromo	1,270,183	A→G	intergenic (-290/-31)	PFL_RS05625 ← / → PFL_RS05630	CaiB/BaiF CoA transferase/ YjfI family protein

Fig 5A, no ile, isolate 1	chromo	1,871,714	T→G	V220G (GTC→GGC)	fliA (PFL_RS08400) →	RNA polymerase sigma factor
Fig 5A, no ile, isolate 1	vector	1,354	A→C	F254L (TTT→TTG)	rhaS ←	
Fig 5A, no ile, isolate 2	vector	911	+C	L128+1bp* (385 nt)	rhaR ←	
Fig 5A, no ile, isolate 2	vector	3,794	G→T	G455C (GGG→GTG)	ilvA →	
Fig 5A, no ile, isolate 3	vector	1,548	A→C	W190G (TGG→GGG)	rhaS ←	
Fig 5A, no ile, isolate 4	vector	2,101	Δ194 bp	$rhaS^{\Delta M1\text{-}H5}/P_{rhaSR}^{\Delta 179bp}$	[rhaS]	
Fig 5A, no ile, isolate 5	chromo	4,109,876	G→C	P90R (CCT→CGT)	uvrC (PFL_RS17990) ←	GacA family transcription factor
Fig 5A, no ile, isolate 5	vector	1,693	Δ150 bp	ΔF92-P141 (274-423 nt)	rhaS ←	
Fig 5A, no ile, isolate 6	chromo	5,831,633	Δ18 bp	coding (438-455/942 nt)	rsmH (PFL_RS25775) ←	16S rRNA methyltransferase
Fig 5A, no ile, isolate 6	vector	611	Δ1,046 bp	$rhaS^{\Delta L154\text{-}Q278}$, $rhaR^{\Delta M1\text{-}F228\text{-}\Delta 1bp*}$	[rhaR]–[rhaS]	
Fig 5A, no ile, isolate 7	vector	1,527	Δ18 bp	ΔD191-Q196, F197V (572-589 nt)	rhaS ←	
Fig 5A, no ile, isolate 8	chromo	1,752,664	(CGGTGA)3→4	Intergenic (+263/+201)	PFL_RS32960 → / ← PFL_RS31825	hypothetical proteins
Fig 5A, no ile, isolate 8	vector	407	Δ8 bp	T294-Δ8bp* (882-889 nt)	rhaR ←	
Fig 5A, no ile, isolate 8	vector	3,439	Δ601 bp	$ilvA^{D335E,\ \Delta G336\text{-}stop515}$, Δ63bp downstream	[relE]–ilvA	
Fig 5A, no ile, isolate 9	chromo	6,598,507	G→C	V88V (GTG→GTC)	PFL_RS29355 →	MltA domain-containing protein
Fig 5A, no ile, isolate 9	vector	1,497	G→A	Q207*stop (CAG→TAG)	rhaS ←	
Fig 5A, no ile, isolate 9	vector	3,550	Δ704 bp	$ilvA^{\Delta D373\text{-}stop515}$, $relE^{\Delta R38\text{-}stop96}$ + Δ277bp downstream	[relE]–ilvA	
Fig 5A, no ile, isolate 10	vector	1,623	Δ13 bp	$rhaS^{\Delta S161\text{-}E164\text{-}\Delta 1bp*}$ (481-493nt)	rhaS ←	
Fig 5A, no ile, isolate 10	vector	3,490	Δ457 bp	$ilvA^{\Delta R353\text{-}N505\text{-}\Delta 1bp*}$	[relE]–[ilvA]	
Fig 5A, +ile, rep 1	vector	1,256	Δ1,891 bp	$\Delta rhaR^{40bp}$ - $ilvA^{712bp}$	[rhaR]–[ilvA]	

Fig 5A, +ile, rep 2	vector	1,515	Δ1,679 bp	ΔrhaS^{601bp} - ilvA^{759bp}	[rhaS]–[ilvA]	
Fig 5A, +ile, rep 3	vector	1,940	Δ1,447 bp	ΔrhaS^{182bp} - ilvA^{946bp}	[rhaS]–[ilvA]	
Fig 5A, +ile, rep 4	vector	970	Δ2,940 bp	ΔrhaR^{325bp} - ilvA^{1475bp}	[rhaR]–[ilvA]	
Fig 5A, +ile, rep 5	vector	917	Δ2,402 bp	ΔrhaR^{379bp} - ilvA^{884bp}	[rhaR]–[ilvA]	
Fig 5A, +ile, rep 6	vector	2,031	Δ1,027 bp	ΔrhaS^{85bp} - ilvA^{623bp}	[rhaS]–[ilvA]	
Fig 5A, +ile, rep 7	vector	7,192	Δ2,901 bp	ΔoriV^{220bp} - ilvA^{256bp}	pRO1600 Rep ← / –	
Fig 5A, +ile, rep 8	vector	970	Δ2,940 bp	ΔrhaR^{325bp} - ilvA^{1475bp}	[rhaR]–[ilvA]	
Fig 5A, +ile, rep 9	vector	1,768	Δ1,107 bp	ΔrhaS^{348bp} - ilvA^{440bp}	[rhaS]–[ilvA]	
Fig 5A, +ile, rep 10	vector	1,052	Δ1,934 bp	ΔrhaR^{244bp} - ilvA^{551bp}	[rhaR]–[ilvA]	

††Sequencing details can be found in **Methods and Materials**. Mutations were located in either the pJUMP-T24 vector or the chromosome. Arrows indicate the ORF direction. * asterisk indicates frameshift

Table S4. Statistical Comparisons.

**Comparison**	**P value#**		**Information**
**Figure 3B - Paired Two-tailed Student's T-test**			
ilvA/relE^{RBS3}: + vs - isoleucine	***	<0.0001	Alpha = 0.05
**Figure 4B - One way ANOVA with Tukey's multiple comparison test**			
ilvA/relE^{RBS3} (- ile) vs. *ilvA/relE*^{RBS3} (+ ile)	****	<0.0001	# of families = 1
ilvA/relE^{RBS3} (- ile) vs. *relE*^{RBS3} (+ ile)	****	<0.0001	# of comparisons per family = 3
ilvA/relE^{RBS3} (+ ile) vs. *relE*^{RBS3} (+ ile)	ns	0.1470	Alpha = 0.05
**Figure 6A - One way ANOVA with Tukey's multiple comparison test**			
parent:parent vs. CB 3:parent	*	0.0355	# of families = 1
parent:parent vs. CB 4:parent	*	0.0239	# of comparisons per family = 6
parent:parent vs. CB 2:parent	**	0.0056	Alpha = 0.05
CB 3:parent vs. CB 4:parent	ns	0.9956	
CB 3:parent vs. CB 2:parent	ns	0.7216	
CB 4:parent vs. CB 2:parent	ns	0.8403	
**Figure 6B - One way ANOVA with Dunnett's multiple comparison test**			
parent vector vs. empty	****	<0.0001	# of families = 2
parent vector vs. vector 3	****	<0.0001	# of comparisons per family = 9
parent vector vs. vector 4	****	<0.0001	Alpha = 0.05
parent vector vs. vector 2	****	<0.0001	
parent vector vs. vector 1	****	<0.0001	
parent vector vs. vector 5	****	<0.0001	
parent vector vs. vector 6	****	<0.0001	

parent vector vs. vector 7	****	<0.0001	
parent vector vs. ΔrhaRΔrhaS	****	<0.0001	
empty vs. parent vector	****	<0.0001	
empty vs. vector 3	**	0.0020	
empty vs. vector 4	**	0.0073	
empty vs. vector 2	****	<0.0001	
empty vs. vector 1	**	0.0055	
empty vs. vector 5	**	0.0034	
empty vs. vector 6	*	0.0113	
empty vs. vector 7	**	0.0023	
empty vs. ΔrhaRΔrhaS	**	0.0045	

[#]Statistical differences were assessed using GraphPad Prism v.9.5.1 software. For the escape ratio experiment in **Fig. 3B**, statistical analysis was performed on the log transformed data.

Supplemental Reference:

1. Halper, S.M., Hossain, A. and Salis, H.M. (2020) Synthesis Success Calculator: Predicting the Rapid Synthesis of DNA Fragments with Machine Learning. *ACS Synth Biol*, **9**, 1563-1571.
